# Supplementary material for: Loss of GCNT2/I-branched glycans enhances melanoma growth and survival
Source: Nat Commun. 2018 Aug 22;9:3368. doi: 10.1038/s41467-018-05795-0 (PMC6105653; doi:10.1038/s41467-018-05795-0)
Supplement: Supplementary file 1 — Supplementary Information [file 41467_2018_5795_MOESM1_ESM.pdf]

Supplementary Information

Loss of GCNT2/I-branched glycans enhances melanoma growth and survival

Sweeney et al.

Supplementary Figures 1-9
Supplementary Tables 1-2

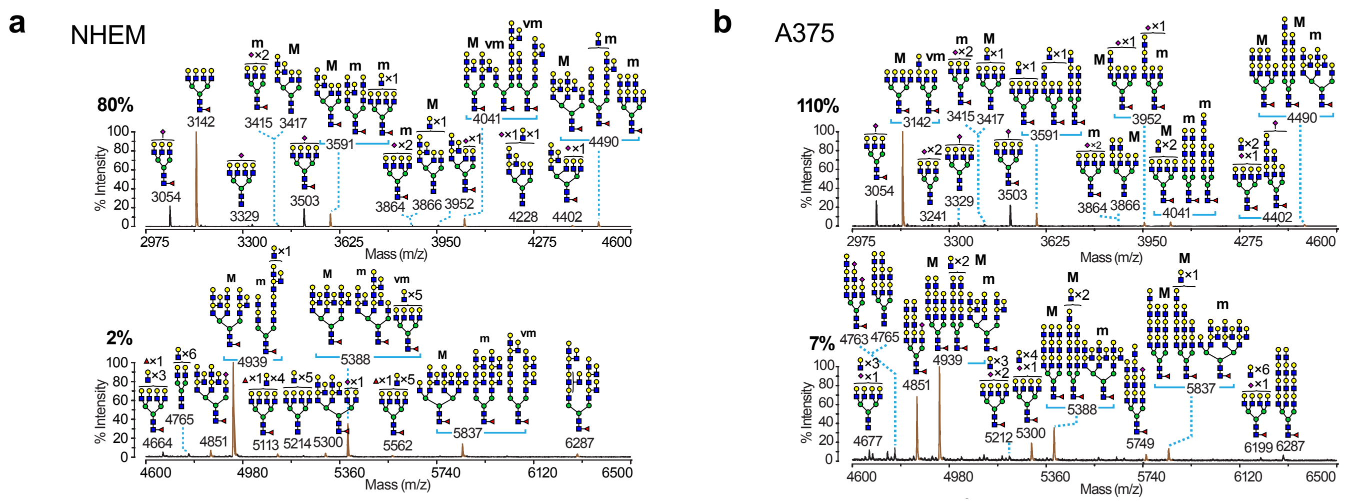

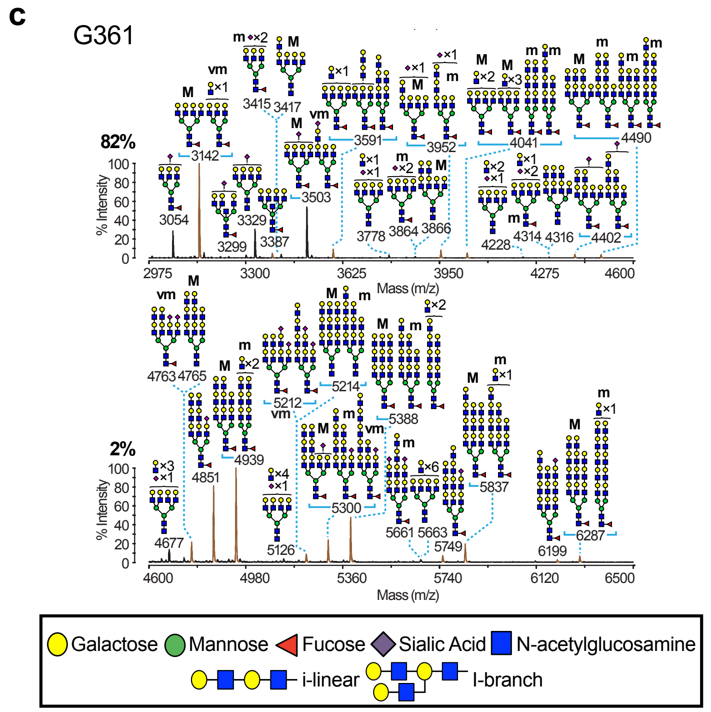

d

Residues	Ret. Time (min)	Fragment ions used for identification (m/z)	Relative abundance (%) NHEM	A375	G361
t-fuc	17.02	102, 115, 118, 131, 162, 175	66.1	65.8	29.3
t-man	18.50	102, 118, 129, 145, 161, 162, 205	257.9	191.3	159.8
t-gal	18.70	102, 118, 129, 145, 161, 162, 205	133.7	176.3	108.5
2-man	19.97	100, 101, 129, 130, 161, 190	304.3	229.7	165.7
3-gal	20.09	101, 118, 129, 143, 161, 174, 203, 217, 234, 245, 277	2.4	18.3	13.3
6-gal	20.59	99, 102, 118, 129, 159, 162, 173, 189, 233	23.9	45.8	35.5
2,4-man	20.97	113, 130, 173, 190, 233	13.4	21.4	18.6
2,6-man	21.37	99, 100, 129, 130, 189, 190, 233, 234	16.9	30.9	32.7
3,6-man	21.55	118, 129, 139, 143, 174, 189, 202, 234	99.8	99.4	98.0
3,6-gal	21.98	118, 129, 139, 143, 174, 189, 202, 234	0.4	0.0	0.0
3,4,6-man	21.98	118, 129, 139	0.2	0.6	2.0
t-GlcNAc	22.33	117, 129, 143, 145, 159, 203, 205	0.2	2.6	4.6
4-GlcNAc	23.34	117, 129, 143, 159, 173, 203, 233	47.0	57.9	104.7
3,4-GlcNAc	24.08	117, 142, 159, 171, 231, 244	0.4	2.0	0.8
4,6-GlcNAc	24.50	117, 143, 159, 203, 261	3.5	7.3	6.5

e

Cell Line	Relative abundance (%)* i-(linear)	I-(Branched)
Normal Human Epidermal Melanocytes	14	86
Immortalized Human Melanocytes	31	69
Short Term Human Melanoma Culture #1	41	59
UACC62 Human Melanoma Cell Line	52	48
Short Term Human Melanoma Culture #2	58	42
Short Term Human Melanoma Culture #3	60	40
A375 Human Melanoma Cell Line	77	23
G361 Human Melanoma Cell Line	79	21

* Classification of all cell lines was based on all ion in the m/z 5000-7000 range, on their *normalized relative intensity* (from MS) and the *percentage of linear/branched ratio* (from MS/MS).
The linear/branched percentage for each cell line, was calculated summing for all the above peaks the [*normalized relative intensity*]*[*% linear/branched ratio*]. The normalized relative intensity was calculated from the MALDI-TOF/TOF MS/MS data corresponding to common linear/branched fragments.

Supplementary Figure 1 N-linked glycan mass spectrometry analysis of normal human epidermal melanocytes (NHEMs) and human melanomas.

(a-c) N-glycan spectral profiles from MALDI-TOF MS analysis of desialylated NHEMs **(a)** and of human melanoma A375 **(b)** or G361 **(c)** cell lines. Cartoon structures were drawn according to http://www.functionalglycomics.org guidelines and are representative from repeat experiments on (2) different biological replicates. **(d)** Table showing relative abundance of different glycan residues for select molecular spectra in NHEM, A375 and G361 melanoma cell lines. **(e)** Table showing relative abundance of i-linear and I-branched glycans for select molecular spectra in NHEM, short-term human melanoma cultures and in human melanoma cell lines.

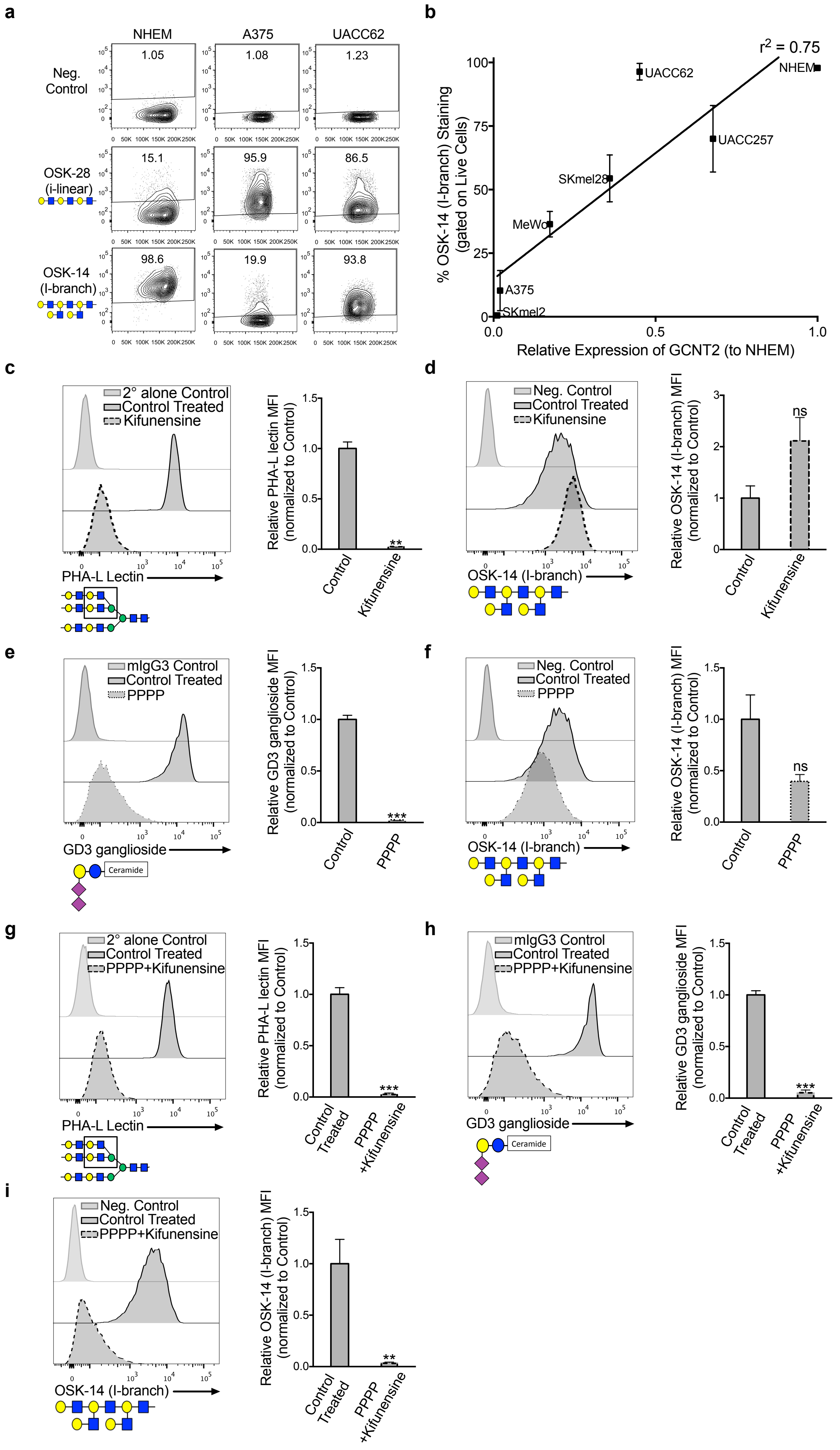

a
NHEM
A375
UACC62
Neg. Control
1.05
1.08
1.23
OSK-28 (i-linear)
15.1
95.9
86.5
OSK-14 (I-branch)
98.6
19.9
93.8
b
r2 = 0.75
% OSK-14 (I-branch) Staining (gated on Live Cells)
Relative Expression of GCNT2 (to NHEM)
UACC62
NHEM
UACC257
SKmel28
MeWo
A375
SKmel2
c
2° alone Control
Control Treated
Kifunensine
PHA-L Lectin
Relative PHA-L lectin MFI (normalized to Control)
Control
Kifunensine
**
d
Neg. Control
Control Treated
Kifunensine
OSK-14 (I-branch)
Relative OSK-14 (I-branch) MFI (normalized to Control)
ns
e
mIgG3 Control
Control Treated
PPPP
GD3 ganglioside
Ceramide
Relative GD3 ganglioside MFI (normalized to Control)
***
f
Neg. Control
Control Treated
PPPP
OSK-14 (I-branch)
Relative OSK-14 (I-branch) MFI (normalized to Control)
ns
g
2° alone Control
Control Treated
PPPP+Kifunensine
PHA-L Lectin
Relative PHA-L lectin MFI (normalized to Control)
Control Treated
PPPP +Kifunensine
***
h
mIgG3 Control
Control Treated
PPPP+Kifunensine
GD3 ganglioside
Ceramide
Relative GD3 ganglioside MFI (normalized to Control)
***
i
Neg. Control
Control Treated
PPPP+Kifunensine
OSK-14 (I-branch)
Relative OSK-14 (I-branch) MFI (normalized to Control)
**

Supplementary Figure 2 N-linked glycoproteins and glycolipids display I-branched glycans on melanoma cells. **(a)** Flow cytometry plots of cell surface i-linear (OSK-28 antibody) and I-branched (OSK-14 antibody) glycan expression on NHEM and on human melanoma A375 and UACC62 cell lines. **(b)** Correlation of GCNT2 gene expression (measured by qRT-PCR – relative to NHEMs) and cell surface expression of I-branched glycans (measured by flow cytometry using OSK-14 antibody). **(c,d)** Representative histogram (left) and quantitation (right) of PHA-L lectin **(c)** and OSK-14 (I-branched) antibody **(d)** staining of control treated and kifunensine treated $GCNT2^+$ UACC62 human melanoma cell line. **(e,f)** Representative histogram (left) and quantitation (right) of GD3 ganglioside **(e)** and OSK-14 (I-branched) antibody **(f)** staining of control treated and kifunensine treated $GCNT2^+$ UACC62 human melanoma cell line. **(g-i)** Representative histogram (left) and quantitation (right) of PHA-L lectin **(g)** GD3 ganglioside **(h)** and OSK-14 (I-branched) antibody **(i)** staining of control treated and kifunensine treated $GCNT2^+$ UACC62 human melanoma cell line. Statistical analyses used were unpaired two-tailed Student's *t* test. For correlation of I-branch glycan expression and GCNT2 gene expression, linear regression was used. Results are representative of n = 3 experiments (mean ± SEM; ** $p < 0.01$; *** $p < 0.001$).

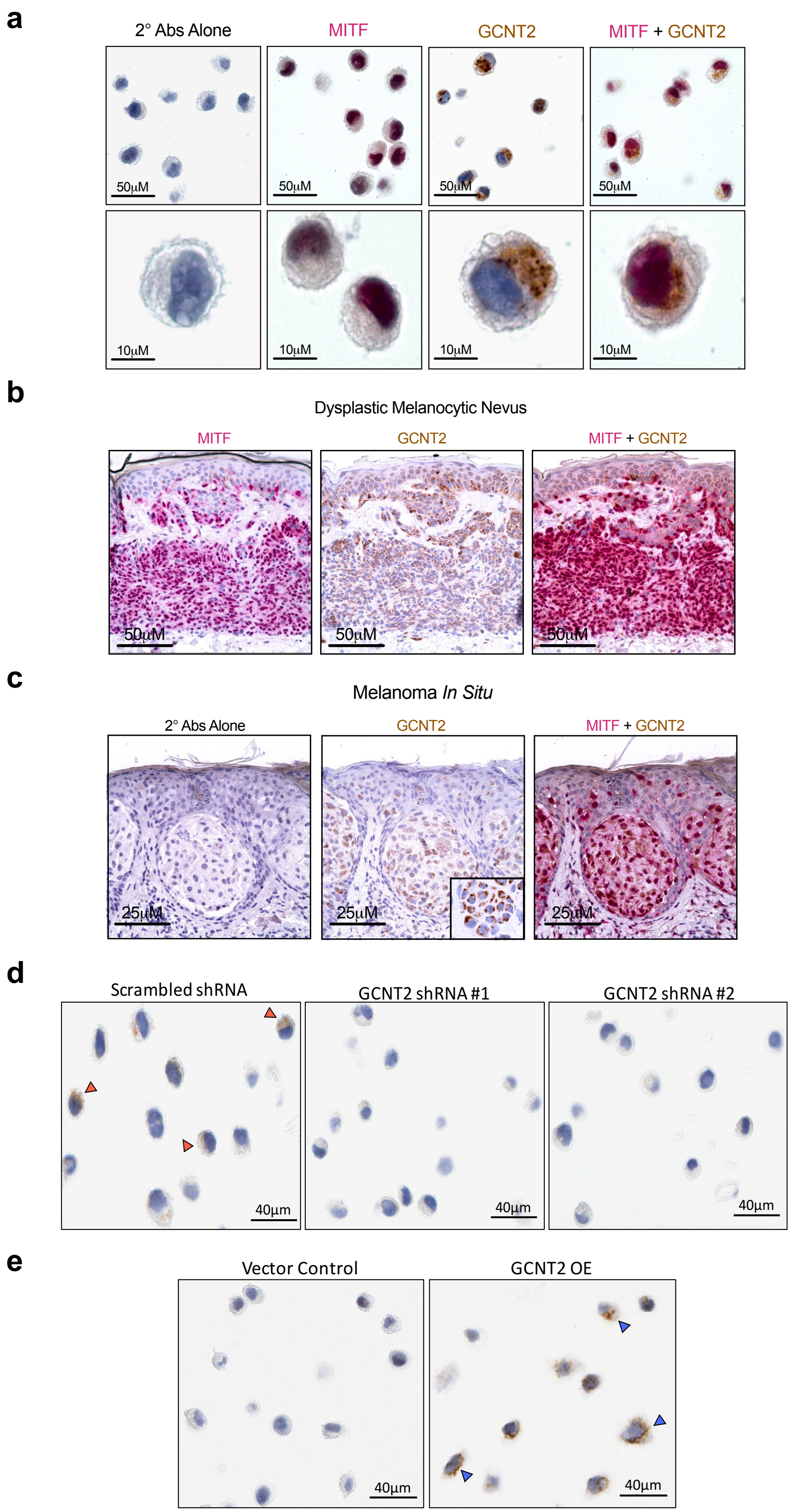

Supplementary Figure 3 GCNT2 expression is downregulated as melanomas progress.

(a-c) Dual immunohistochemistry (IHC) of vector control and GCNT2 overexpressing cell variants **(a)**, a human dysplastic nevus **(b)** and a melanoma *in situ* **(c)** stained for MITF and GCNT2. Photomicrographs were taken at 10x or at 40X. **(d,e)** IHC of GCNT2 expression in UACC62 scrambled control and GCNT2 KD **(d)** cell variants and A375 vector control and GCNT2 OE **(e)** cell variants. Photographs were taken at 40X.

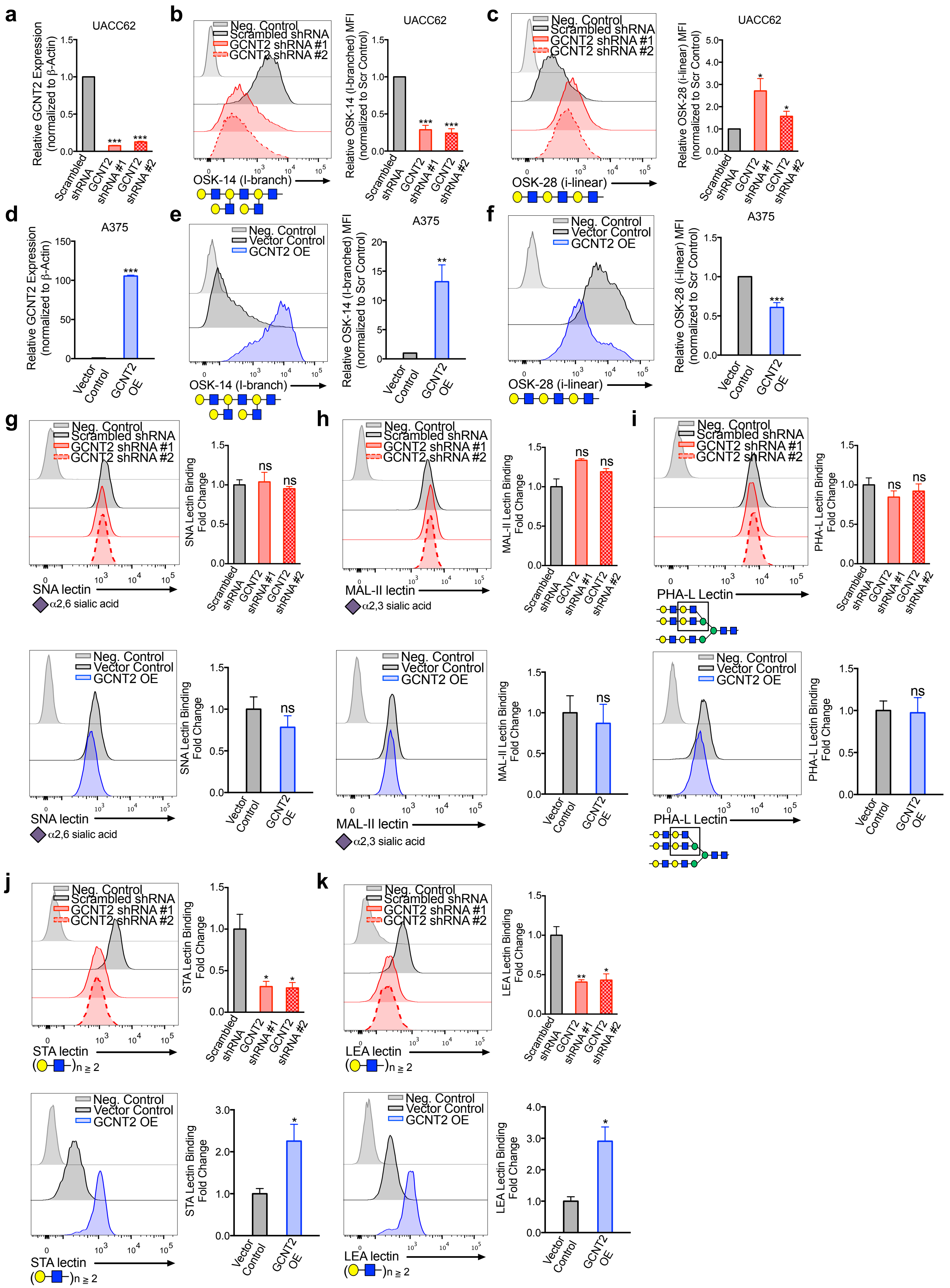
a
UACC62
Relative GCNT2 Expression (normalized to β-Actin)
Scrambled shRNA
GCNT2 shRNA #1
GCNT2 shRNA #2
b
Neg. Control
Scrambled shRNA
GCNT2 shRNA #1
GCNT2 shRNA #2
OSK-14 (I-branch)
Relative OSK-14 (I-branched) MFI (normalized to Scr Control)
c
OSK-28 (i-linear)
Relative OSK-28 (i-linear) MFI (normalized to Scr Control)
d
A375
Vector Control
GCNT2 OE
e
f
g
SNA lectin
α2,6 sialic acid
SNA Lectin Binding Fold Change
ns
h
MAL-II lectin
α2,3 sialic acid
MAL-II Lectin Binding Fold Change
i
PHA-L Lectin
PHA-L Lectin Binding Fold Change
j
STA lectin
STA Lectin Binding Fold Change
k
LEA lectin
LEA Lectin Binding Fold Change

Supplementary Figure 4 I-branched glycans are decreased in GCNT2 knockdown (KD) and increased in GCNT2 overexpressing (OE) melanoma cell variants.

(a) qRT-PCR of GCNT2 gene expression in UACC62 scrambled control and GCNT2-knockdown (KD) cell variants. **(b)** Representative flow cytometry plot (left) and quantitation (right) of cell surface I-branched glycan expression on UACC62 scrambled control and GCNT2 KD cell variants. **(c)** Representative flow cytometry plot (left) and quantitation (right) of cell surface i-linear glycan expression on UACC62 scrambled control and GCNT2 KD cell variants. **(d)** qRT-PCR of GCNT2 gene expression in A375 vector control and GCNT2-overexpressing (OE) cell variants. **(e)** Representative flow cytometry plot (left) and quantitation (right) of cell surface I-branched glycan expression on A375 vector control and GCNT2 OE cell variants. **(f)** Representative flow cytometry plot (left) and quantitation (right) of cell surface i-linear glycan expression on A375 vector control and GCNT2 OE cell variants. **(g)** Representative histograms (left) and quantitation (right) of SNA lectin binding to UACC62 control and GCNT2 KD (top) cell variants and A375 vector control and GCNT2 OE (bottom) cell variants. **(h)** Representative histograms (left) and quantitation (right) of MAL-II lectin binding to UACC62 control and GCNT2 KD (top) cell variants and A375 vector control and GCNT2 OE (bottom) cell variants. **(i)** Representative histograms (left) and quantitation (right) of PHA-L lectin binding to UACC62 control and GCNT2 KD (top) cell variants and A375 vector control and GCNT2 OE (bottom) cell variants. **(j)** Representative histograms (left) and quantitation (right) of STA lectin binding to UACC62 control and GCNT2 KD (top) cell variants and A375 vector control and GCNT2 OE (bottom) cell variants. **(k)** Representative histograms (left) and quantitation (right) of LEA lectin binding to UACC62 control and GCNT2 KD (top) cell variants and A375 vector control and GCNT2 OE (bottom) cell variants. Statistical analyses used were unpaired two-tailed Student's *t* test. Results are representative of $n = 3$-4 experiments (mean ± SEM; *$p < 0.05$; **$p < 0.01$; ***$p < 0.001$).

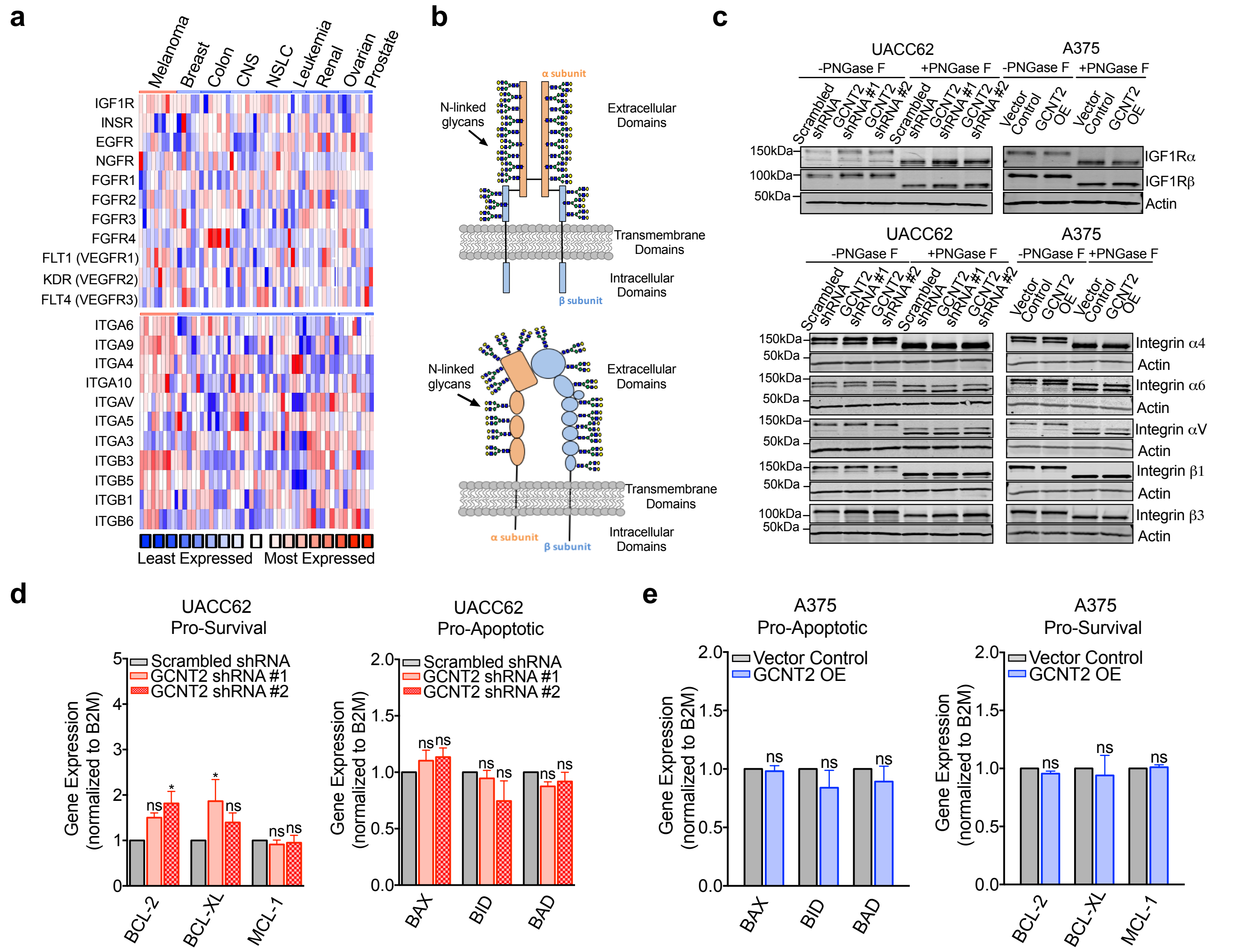

Supplementary Figure 5 Insulin-like growth factor receptor (IGF1R) and various integrin α and β chains are heavily N-glycosylated.

(a) Heatmap of growth factor receptor and various integrin α and β chain gene expression in melanomas versus eight other common malignancies. Generated using the Oncomine (www.oncomine.com) Compendia Cell Line panel. **(b)** Schematic representation of IGF1R and integrin α6β1 heterodimer with predicted N-linked glycan sites. **(c)** Immunoblots of UACC62 control and GCNT2 KD (left) and A375 control and GCNT2 OE (right) lysates treated with Peptide N-Glycosidase F (PNGase F) and blotted with IGF1Rα/β chain antibodies (top) or with Integrin α4, α6, αV, β1 and β3 antibodies (bottom). Actin used as loading control. **(d,e)** Gene expression of apoptotic and survival family genes measured by qRT-PCR in UACC62 control and GCNT2 KD **(d)** and A375 vector control and GCNT2 OE **(e)** cell variants plated on plastic tissue culture grade plates. Statistical analyses used were one-way ANOVA with Dunnett's multiple comparisons test or unpaired two-tailed Student's *t* test. Results are representative of n = 3 experiments (mean ± SEM; $^*p < 0.05$).

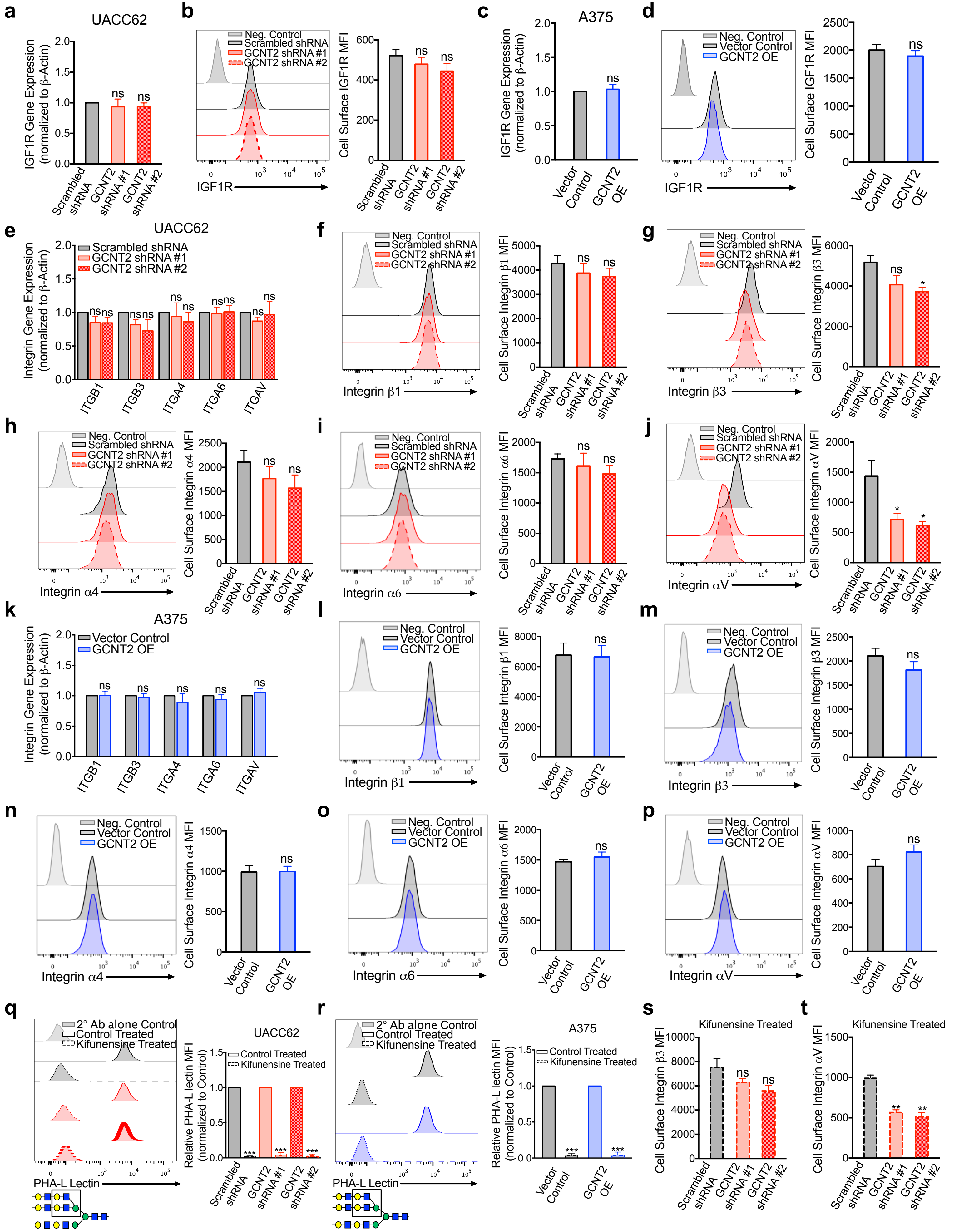
a
UACC62
IGF1R Gene Expression (normalized to β-Actin)
Scrambled shRNA
GCNT2 shRNA #1
GCNT2 shRNA #2
ns
b
Neg. Control
Scrambled shRNA
GCNT2 shRNA #1
GCNT2 shRNA #2
IGF1R
Cell Surface IGF1R MFI
c
A375
IGF1R Gene Expression (normalized to β-Actin)
Vector Control
GCNT2 OE
d
Neg. Control
Vector Control
GCNT2 OE
IGF1R
Cell Surface IGF1R MFI
e
UACC62
Integrin Gene Expression (normalized to β-Actin)
ITGB1
ITGB3
ITGA4
ITGA6
ITGAV
f
Integrin β1
Cell Surface Integrin β1 MFI
g
Integrin β3
Cell Surface Integrin β3 MFI
h
Integrin α4
Cell Surface Integrin α4 MFI
i
Integrin α6
Cell Surface Integrin α6 MFI
j
Integrin αV
Cell Surface Integrin αV MFI
k
A375
Integrin Gene Expression (normalized to β-Actin)
l
Integrin β1
Cell Surface Integrin β1 MFI
m
Integrin β3
Cell Surface Integrin β3 MFI
n
Integrin α4
Cell Surface Integrin α4 MFI
o
Integrin α6
Cell Surface Integrin α6 MFI
p
Integrin αV
Cell Surface Integrin αV MFI
q
2° Ab alone Control
Control Treated
Kifunensine Treated
PHA-L Lectin
UACC62
Relative PHA-L lectin MFI (normalized to Control)
r
A375
PHA-L Lectin
Relative PHA-L lectin MFI (normalized to Control)
s
Kifunensine Treated
Cell Surface Integrin β3 MFI
t
Kifunensine Treated
Cell Surface Integrin αV MFI

Supplementary Figure 6 GCNT2 cell variants express similar cell surface levels of IGF1R and integrins α4, α6, β1 and β3.

(a) IGF1R gene expression measured by qRT-PCR in UACC62 control and GCNT2 KD cell variants. **(b)** Representative histogram (left) and quantitation (right) of cell surface IGF1R expression in UACC62 control and GCNT2 KD cell variants. **(c)** IGF1R gene expression measured by qRT-PCR in A375 control and GCNT2 OE cell variants. **(d)** Representative histogram (left) and quantitation (right) of cell surface IGF1R expression in A375 control and GCNT2 OE cell variants. **(e)** Integrins β1, β3, α4, α6, and αV and gene expression measured by qRT-PCR in UACC62 control and GCNT2 KD cell variants. **(f-j)** Representative histogram (left) and quantitation (right) of cell surface Integrin β1 **(f)**, Integrin β3 **(g)**, Integrin α4 **(h)**, Integrin α6 **(i)** and Integrin αV **(j)** expression on UACC62 control and GCNT2 KD cell variants. **(k)** Integrins β1, β3, α4, α6, and αV and gene expression measured by qRT-PCR in A375 control and GCNT2 OE cell variants. **(l-p)** Representative histogram (left) and quantitation (right) of cell surface Integrin β1 **(l)**, Integrin β3 **(m)**, Integrin α4 **(n)**, Integrin α6 **(o)** and Integrin αV **(p)** expression on A375 control and GCNT2 OE cell variants. **(q)** Representative flow cytometry plot (left) and quantitation (right) of PHA-L lectin staining of UACC62 control and GCNT2 KD cell variants treated with kifunensine. **(r)** Representative flow cytometry plot (left) and quantitation (right) of PHA-L lectin staining of A375 control and GCNT2 OE cell variants treated with kifunensine. **(s,t)** Quantitation of cell surface Integrin β3 **(s)** and Integrin αV **(t)** expression in kifunensine treated UACC62 control and GCNT2 KD cell variants. Statistical analyses used were one-way ANOVA with Dunnett's multiple comparisons test or unpaired two-tailed Student's *t* test. Results representative of n = 3 experiments (mean ± SEM; *p<0.05; **p< 0.01; ***p < 0.001).

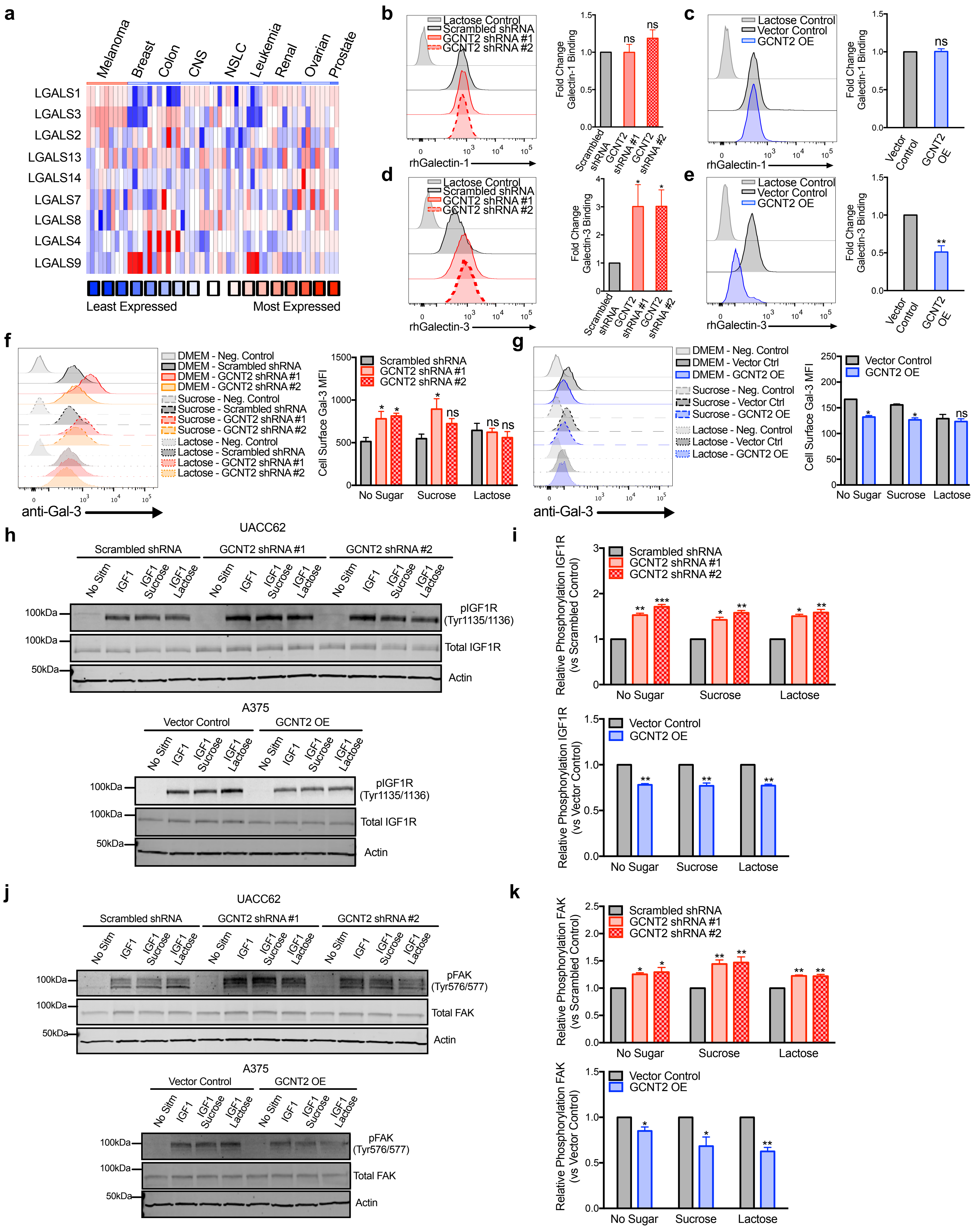
a
Melanoma
Breast
Colon
CNS
NSLC
Leukemia
Renal
Ovarian
Prostate
LGALS1
LGALS3
LGALS2
LGALS13
LGALS14
LGALS7
LGALS8
LGALS4
LGALS9
Least Expressed
Most Expressed
b
Lactose Control
Scrambled shRNA
GCNT2 shRNA #1
GCNT2 shRNA #2
rhGalectin-1
Fold Change Galectin-1 Binding
ns
ns
Scrambled shRNA
GCNT2 shRNA #1
GCNT2 shRNA #2
c
Lactose Control
Vector Control
GCNT2 OE
rhGalectin-1
Fold Change Galectin-1 Binding
ns
Vector Control
GCNT2 OE
d
Lactose Control
Scrambled shRNA
GCNT2 shRNA #1
GCNT2 shRNA #2
rhGalectin-3
Fold Change Galectin-3 Binding
e
Lactose Control
Vector Control
GCNT2 OE
rhGalectin-3
Fold Change Galectin-3 Binding
f
DMEM - Neg. Control
DMEM - Scrambled shRNA
DMEM - GCNT2 shRNA #1
DMEM - GCNT2 shRNA #2
Sucrose - Neg. Control
Sucrose - Scrambled shRNA
Sucrose - GCNT2 shRNA #1
Sucrose - GCNT2 shRNA #2
Lactose - Neg. Control
Lactose - Scrambled shRNA
Lactose - GCNT2 shRNA #1
Lactose - GCNT2 shRNA #2
anti-Gal-3
Cell Surface Gal-3 MFI
Scrambled shRNA
GCNT2 shRNA #1
GCNT2 shRNA #2
No Sugar
Sucrose
Lactose
g
DMEM - Neg. Control
DMEM - Vector Ctrl
DMEM - GCNT2 OE
Sucrose - Neg. Control
Sucrose - Vector Ctrl
Sucrose - GCNT2 OE
Lactose - Neg. Control
Lactose - Vector Ctrl
Lactose - GCNT2 OE
anti-Gal-3
Cell Surface Gal-3 MFI
Vector Control
GCNT2 OE
No Sugar
Sucrose
Lactose
h
UACC62
Scrambled shRNA
GCNT2 shRNA #1
GCNT2 shRNA #2
No Sitm
IGF1
IGF1 Sucrose
IGF1 Lactose
100kDa
pIGF1R (Tyr1135/1136)
Total IGF1R
50kDa
Actin
A375
Vector Control
GCNT2 OE
i
Relative Phosphorylation IGF1R (vs Scrambled Control)
Relative Phosphorylation IGF1R (vs Vector Control)
j
pFAK (Tyr576/577)
Total FAK
k
Relative Phosphorylation FAK (vs Scrambled Control)
Relative Phosphorylation FAK (vs Vector Control)

Supplementary Figure 7 GCNT2 inhibits Galectin-3 (Gal-3)-binding but Gal-3 does not regulate differential IGF1R or FAK signaling responses in melanoma cells.

(a) Heatmap of various galectin gene expression in melanomas versus eight other common malignancies. Generated using the Oncomine (www.oncomine.com) Compendia Cell Line panel. **(b,c)** Representative histogram (left) and quantitation (right) of rhGal-1-binding to UACC62 control and GCNT2 KD **(b)** and A375 control and GCNT2 OE **(c)** cell variants. **(d,e)** Representative histogram (left) and quantitation (right) of rhGal-3-binding to UACC62 control and GCNT2 KD **(d)** and A375 control and GCNT2 OE **(e)** cell variants. **(f,g)** Representative histogram (left) and quantitation (right) of endogenous Gal-3-binding to UACC62 control and GCNT2 KD **(f)** and A375 control and GCNT2 OE **(g)** cell variants incubated for 24hrs in DMEM supplemented with No Sugar, 50mM Sucrose or 50mM Lactose. **(h,i)** Immunoblots **(h)** and quantitation **(i)** of phosphorylated and total IGF1R in UACC62 control and GCNT2 KD (top) and A375 control and GCNT2 OE (bottom) cell variants incubated for 24hrs in DMEM supplemented with No Sugar, 50mM Sucrose or 50mM Lactose and then treated with rhIGF-1 for 60 min. **(j,k)** Immunoblots **(j)** and quantitation **(k)** of phosphorylated and total focal adhesion kinase (FAK) in UACC62 control and GCNT2 KD (top) and A375 control and GCNT2 OE (bottom) cell variants incubated for 24hrs in DMEM supplemented with No Sugar, 50mM Sucrose or 50mM Lactose and then plated on ECM for 60 min. Quantitation of phosphorylated proteins was normalized to total protein expression and then compared to control cells. Statistical analyses used were one-way ANOVA with Dunnett’s multiple comparisons test or unpaired two-tailed Student’s *t* test. Results representative of n = 3 experiments (mean ± SEM; *$p<0.05$; **$p< 0.01$).

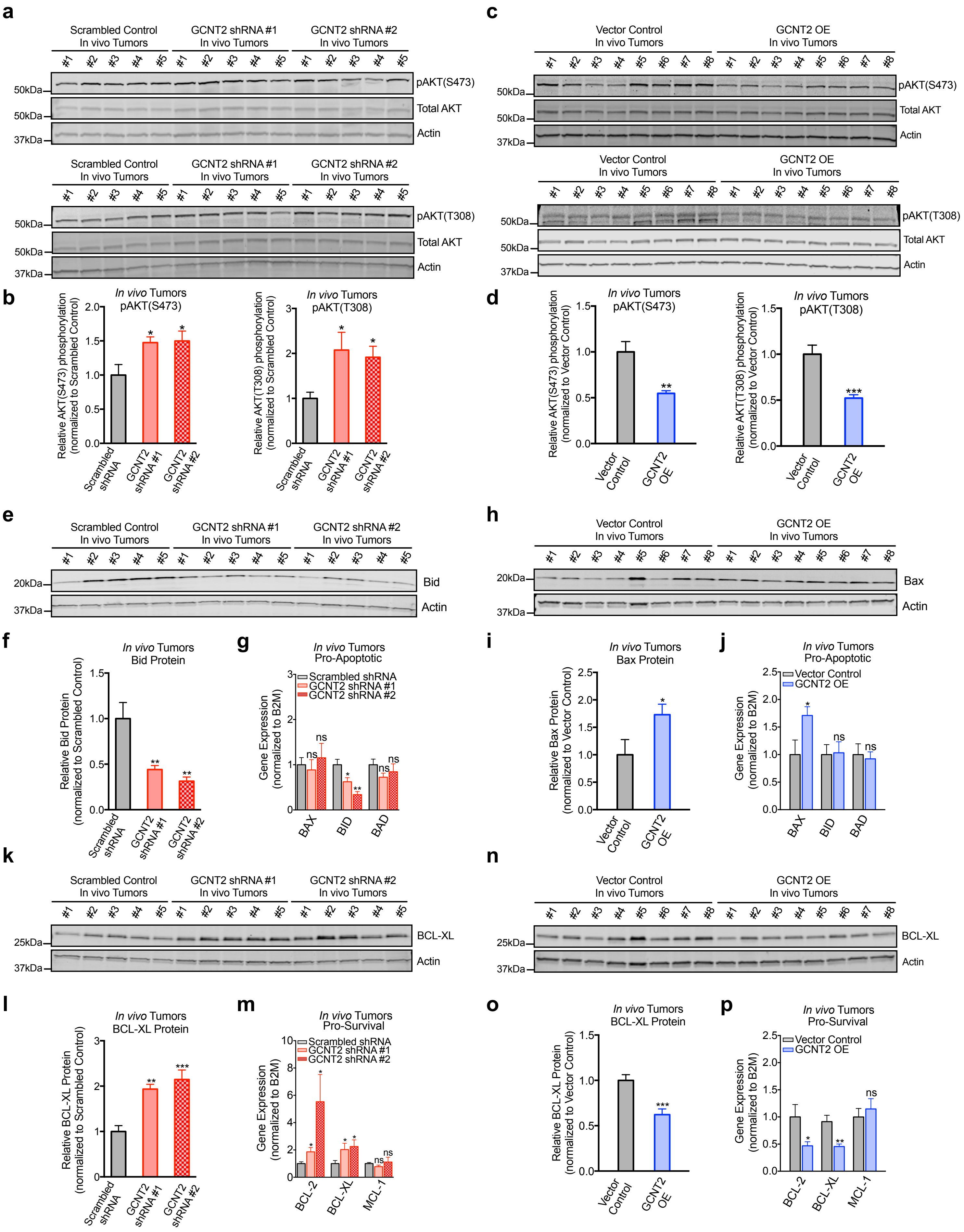
a
Scrambled Control In vivo Tumors
GCNT2 shRNA #1 In vivo Tumors
GCNT2 shRNA #2 In vivo Tumors
#1 #2 #3 #4 #5
50kDa
37kDa
pAKT(S473)
Total AKT
Actin
pAKT(T308)
b
In vivo Tumors pAKT(S473)
Relative AKT(S473) phosphorylation (normalized to Scrambled Control)
In vivo Tumors pAKT(T308)
Relative AKT(T308) phosphorylation (normalized to Scrambled Control)
Scrambled shRNA
GCNT2 shRNA #1
GCNT2 shRNA #2
c
Vector Control In vivo Tumors
GCNT2 OE In vivo Tumors
#1 #2 #3 #4 #5 #6 #7 #8
d
In vivo Tumors pAKT(S473)
Relative AKT(S473) phosphorylation (normalized to Vector Control)
In vivo Tumors pAKT(T308)
Relative AKT(T308) phosphorylation (normalized to Vector Control)
Vector Control
GCNT2 OE
e
20kDa
Bid
h
Bax
f
In vivo Tumors Bid Protein
Relative Bid Protein (normalized to Scrambled Control)
g
In vivo Tumors Pro-Apoptotic
Gene Expression (normalized to B2M)
BAX
BID
BAD
i
In vivo Tumors Bax Protein
Relative Bax Protein (normalized to Vector Control)
j
In vivo Tumors Pro-Apoptotic
k
25kDa
BCL-XL
n
l
In vivo Tumors BCL-XL Protein
Relative BCL-XL Protein (normalized to Scrambled Control)
m
In vivo Tumors Pro-Survival
BCL-2
BCL-XL
MCL-1
o
In vivo Tumors BCL-XL Protein
Relative BCL-XL Protein (normalized to Vector Control)
p
In vivo Tumors Pro-Survival
ns

Supplementary Figure 8 AKT signaling and pro-survival genes are increased in *in vivo* tumors with low GCNT2/ I-branched glycan expression.

(a,b) Immunoblots **(a)** and quantitation **(b)** of serine and threonine AKT phosphorylation from UACC62 control and GCNT2 KD *in vivo* tumors lysates. **(c,d)** Immunoblots **(c)** and quantitation **(d)** of serine and threonine AKT phosphorylation from A375 control and GCNT2 OE *in vivo* tumors lysates. **(e-g)** Immunoblot **(e)** quantitation **(f)** and gene expression **(g)** of pro-apoptotic family member, Bid in UACC62 control and GCNT2 KD *in vivo* tumors. **(h-j)** Immunoblot **(h)**, quantitation **(i)** and gene expression **(j)** of pro-apoptotic family member, Bax in A375 control and GCNT2 OE *in vivo* tumors. **(k-m)** Immunoblot **(k)** quantitation **(l)** and gene expression **(m)** of pro-survival family member, BCL-XL in UACC62 control and GCNT2 KD *in vivo* tumors. **(n-p)** Immunoblot **(n)** quantitation **(o)** and gene expression **(p)** of pro- survival family member, BCL-XL in A375 control and GCNT2 OE *in vivo* tumors. Statistical analyses used were one-way ANOVA with Dunnett's multiple comparisons test or unpaired two-tailed Student's *t* test. Results representative of n = 5-8 tumors per group (mean ± SEM; $*p < 0.05$; $**p < 0.01$; $***p < 0.001$).

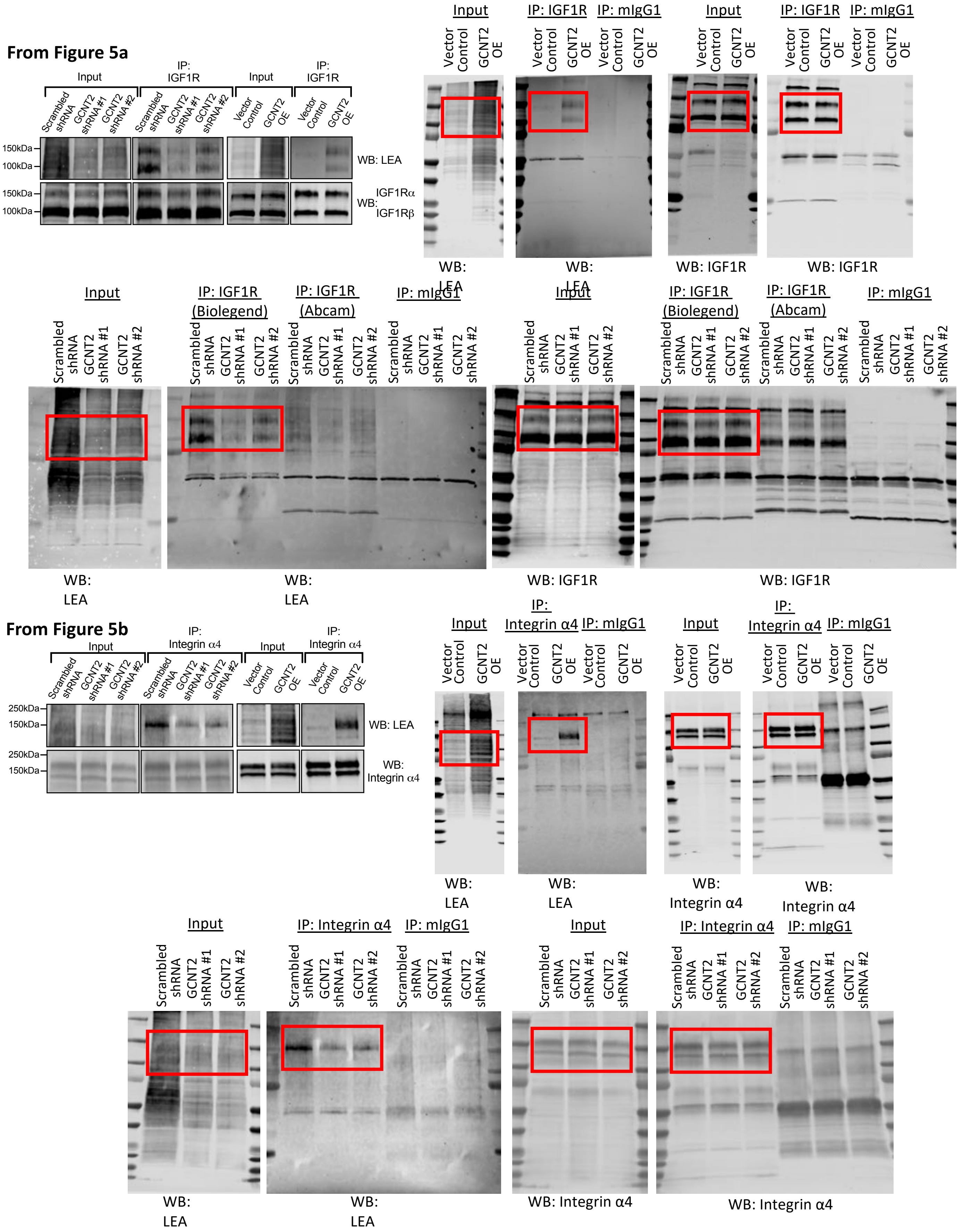

Supplementary Figure 9. Uncropped blot images From Figure 5a and 5b

From Figure 5c

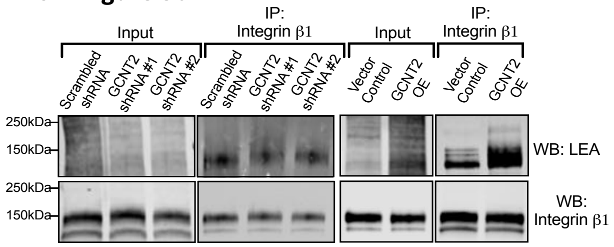

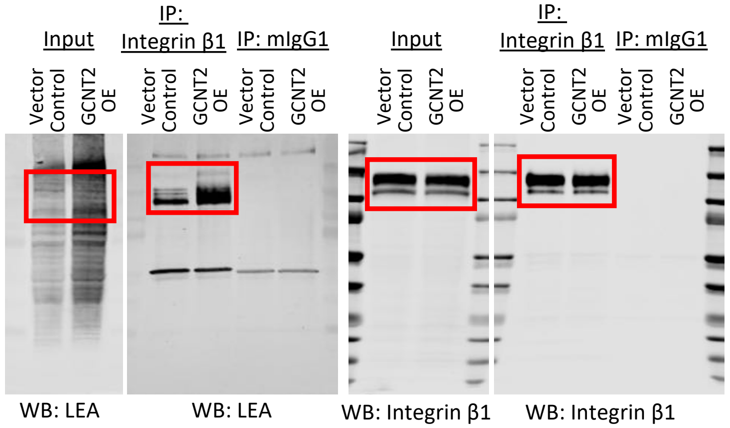

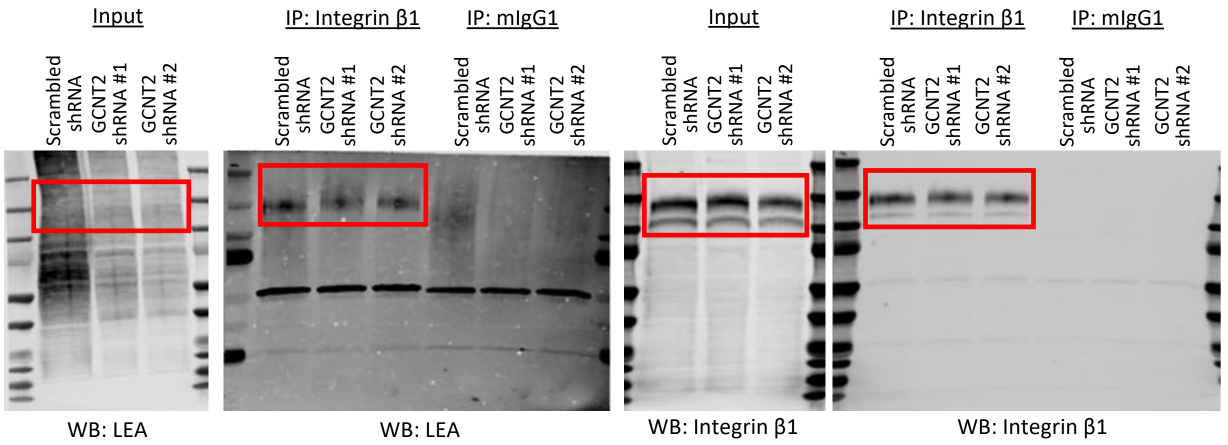

From Figure 5d

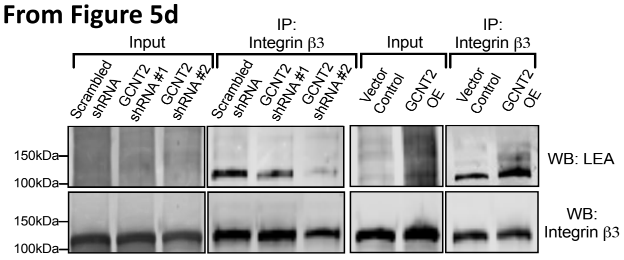

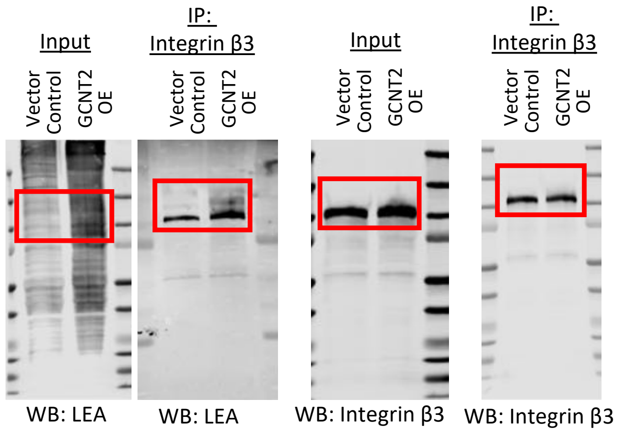

NOTE: Isotype for Integrin β3 is same as Integrin β1 (mIgG1) – all Integrin β3 and Integrin β1 samples IP'd from same lysate prep. and westerns run on same day

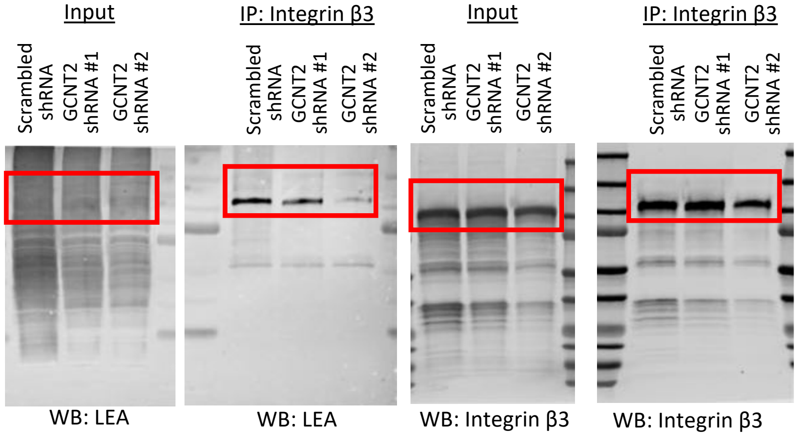

Supplementary Figure 9 (continued). Uncropped blot images From Figure 5c and 5d

From Figure 5h

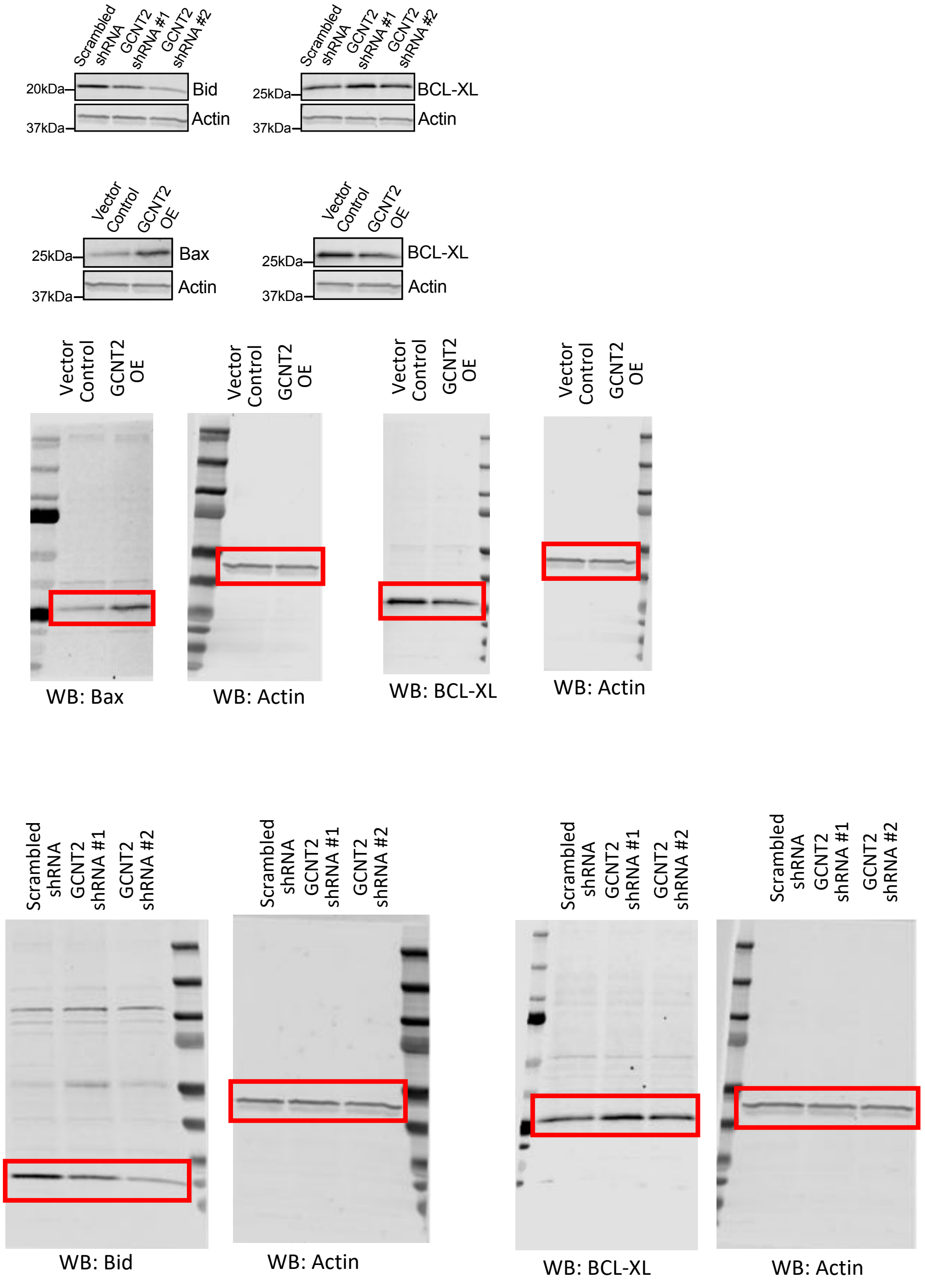

Supplementary Figure 9 (continued). Uncropped blot images From Figure 5h

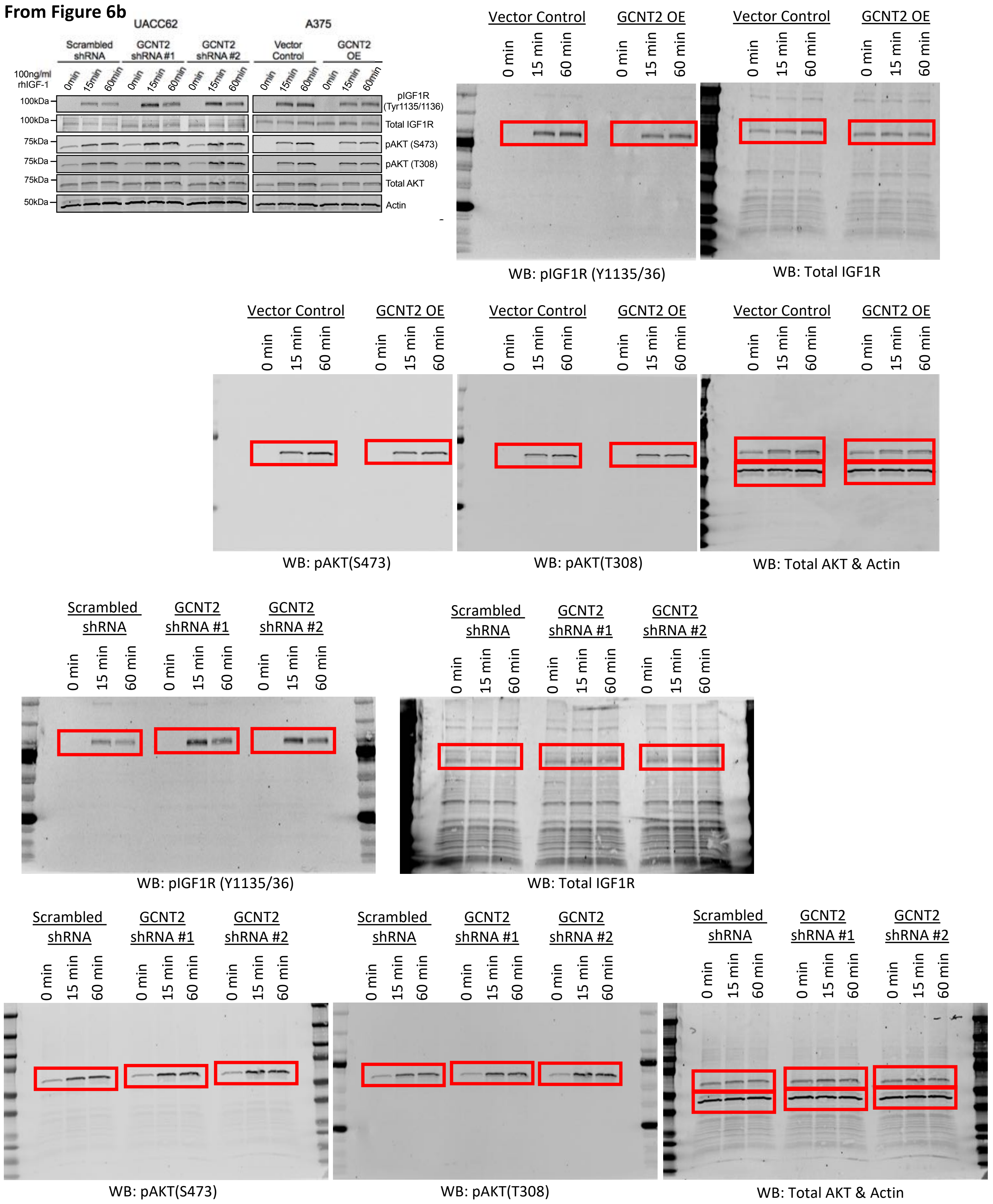

Supplementary Figure 9 (continued). Uncropped blot images From Figure 6b

From Figure 6e

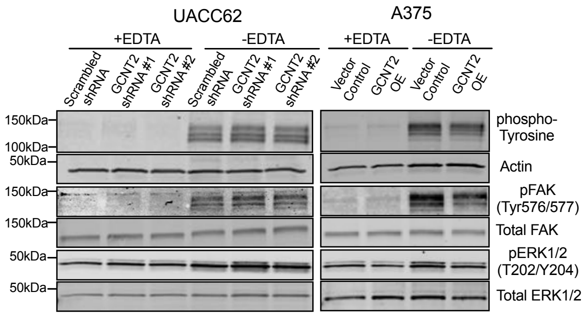

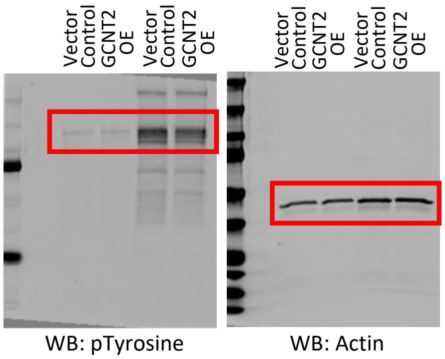

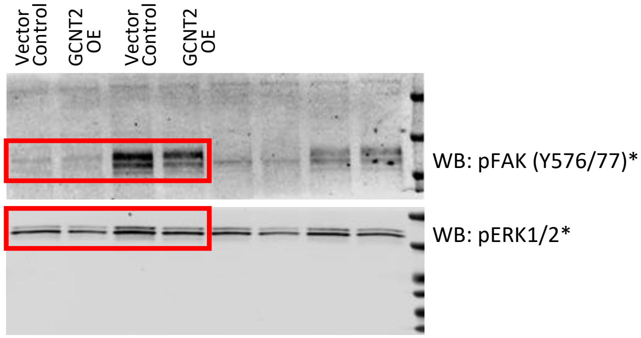

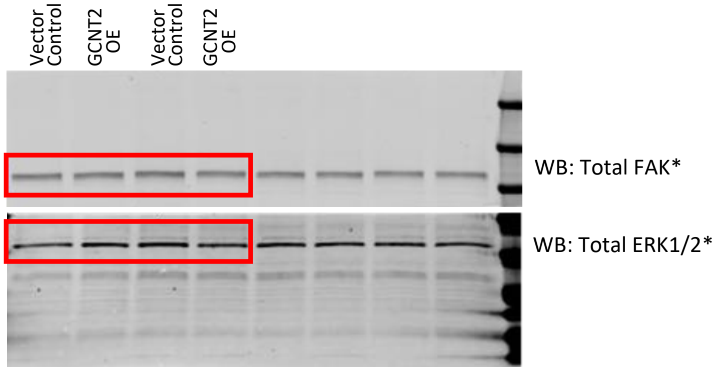

*NOTE: Blots for FAK & ERK1/2 signaling were cut horizontally and blotted separately
(these are the two horizonal pieces from a single blot)

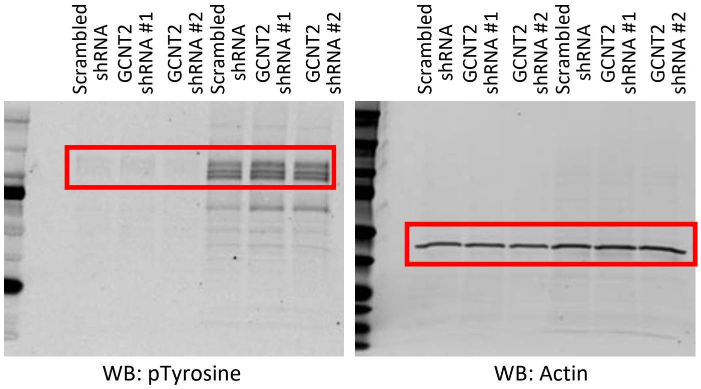

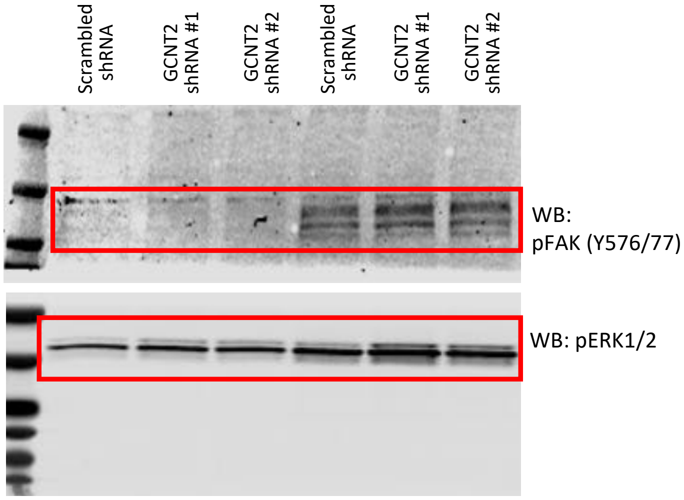

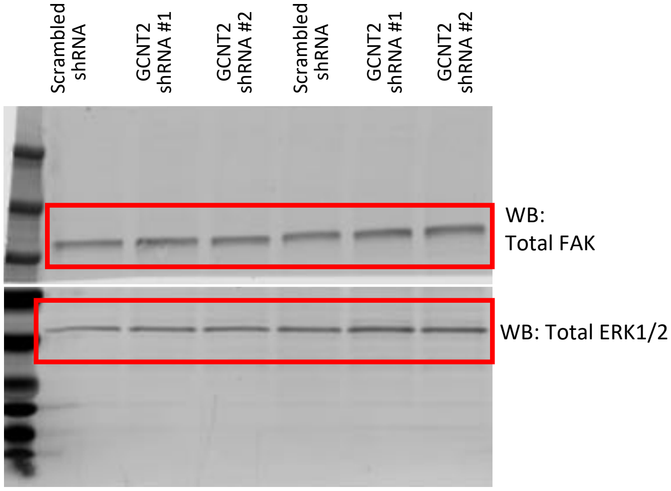

Supplementary Figure 9 (continued). Uncropped blot images From Figure 6e

From Figure 6h

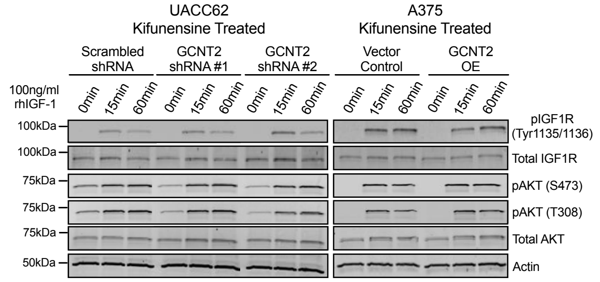

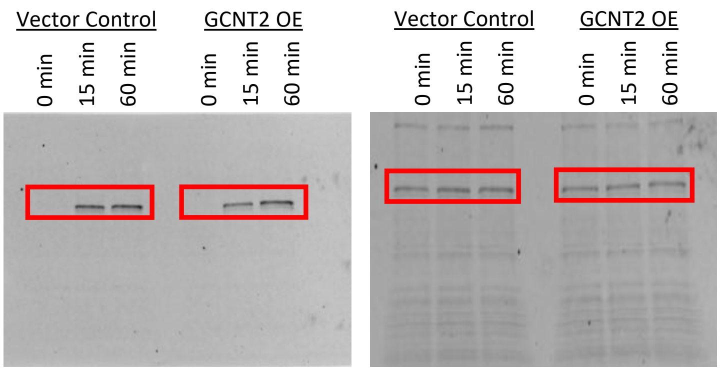

WB: pIGF1R (Y1135/36)

WB: Total IGF1R

*These A375 kifunensine treated cells were run on the right side of the gel/membrane as the H2O treated cells in Figure 6e – See previous blots for molecular weight markers, etc.

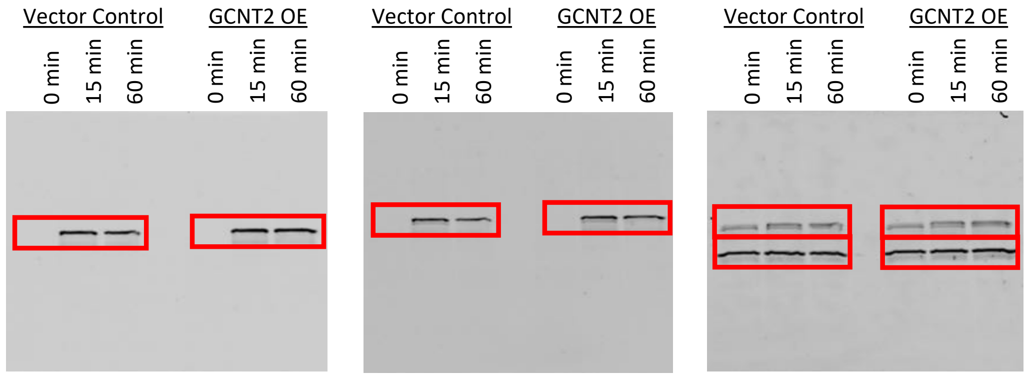

WB: pAKT(S473)

WB: pAKT(T308)

WB: Total AKT & Actin

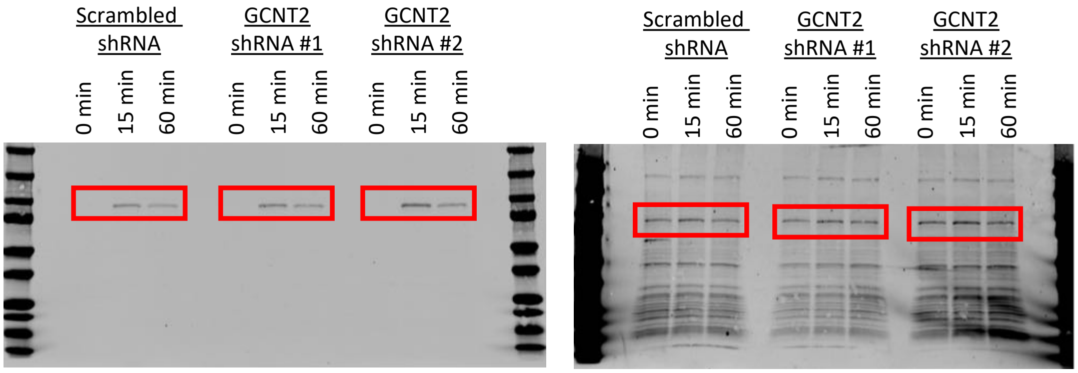

WB: pIGF1R (Y1135/36)

WB: Total IGF1R

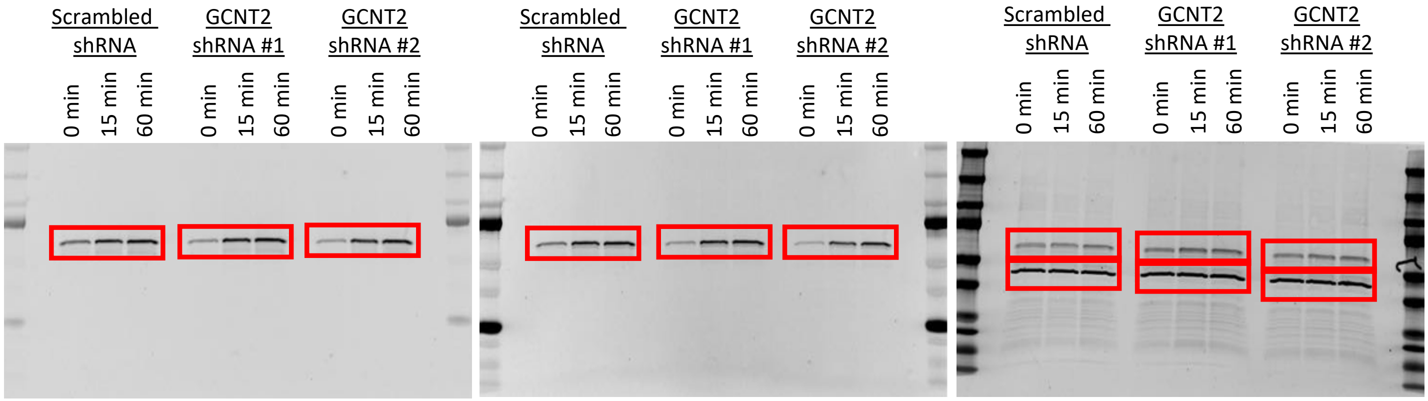

WB: pAKT(S473)

WB: pAKT(T308)

WB: Total AKT & Actin

Supplementary Figure 9 (continued). Uncropped blot images From Figure 6h

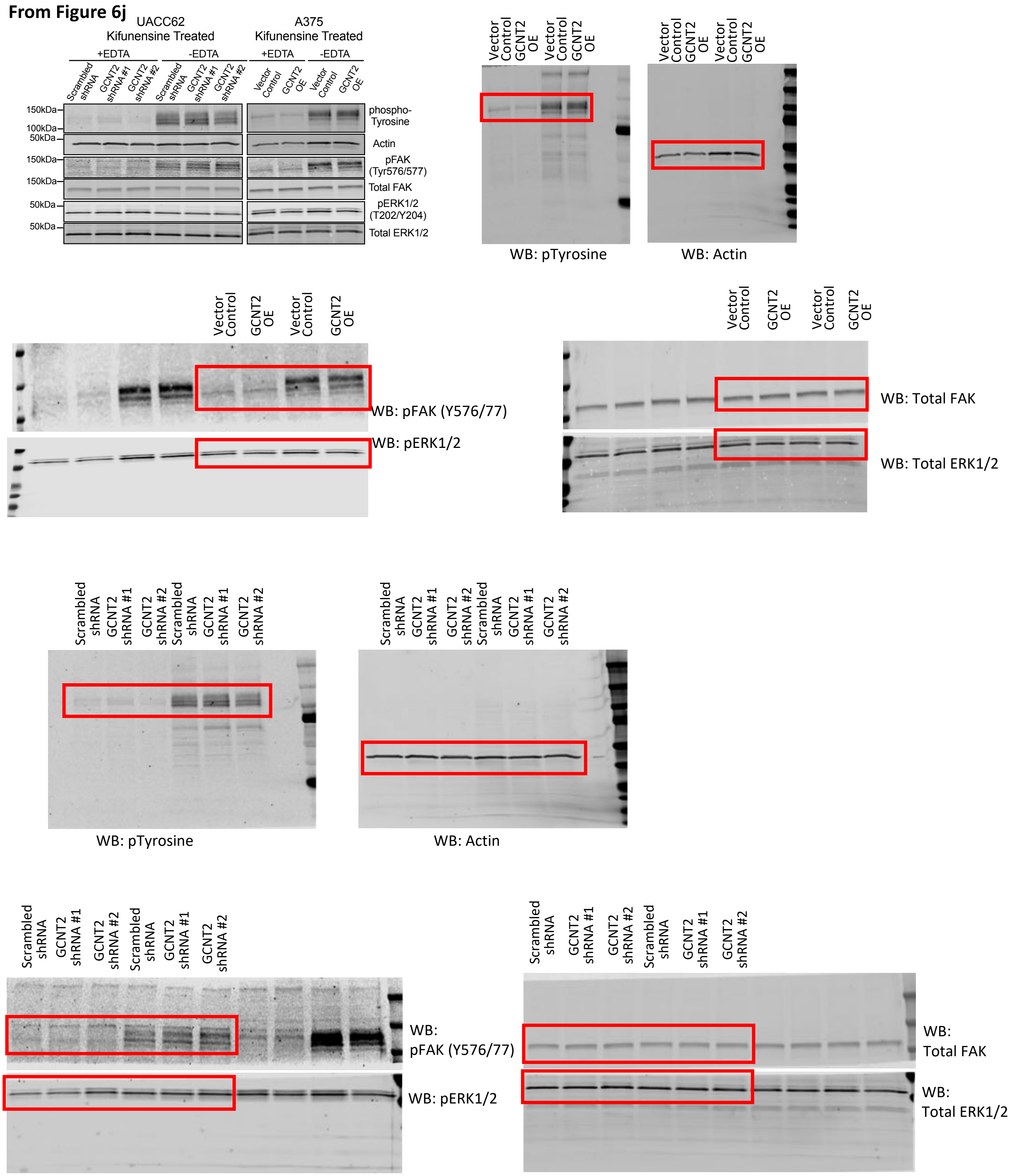

Supplementary Figure 9 (continued). Uncropped blot images From Figure 6j

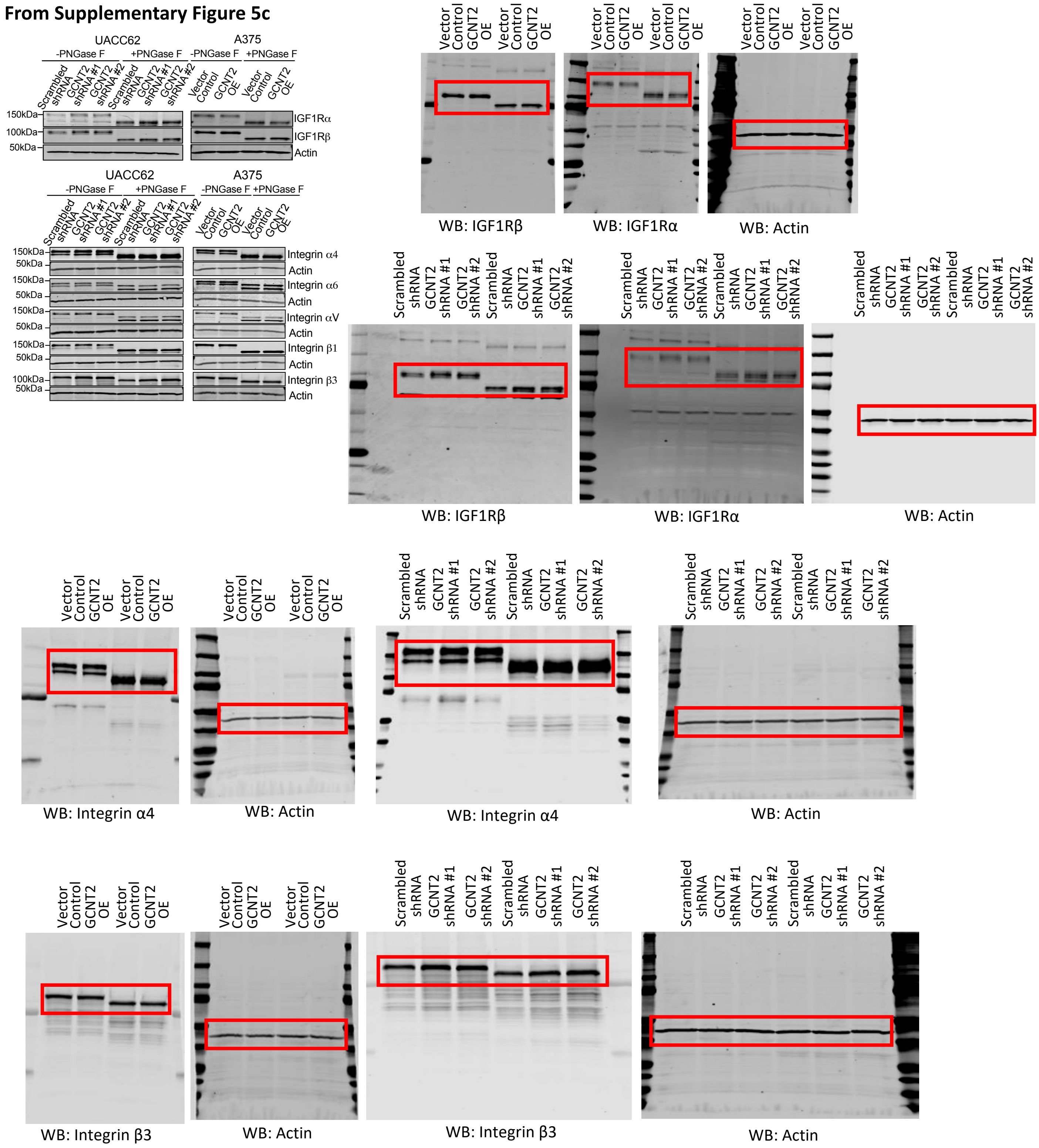

Supplementary Figure 9 (continued). Uncropped blot images From Supplemental Figure 5c

From Supplementary Figure 5c (continued)

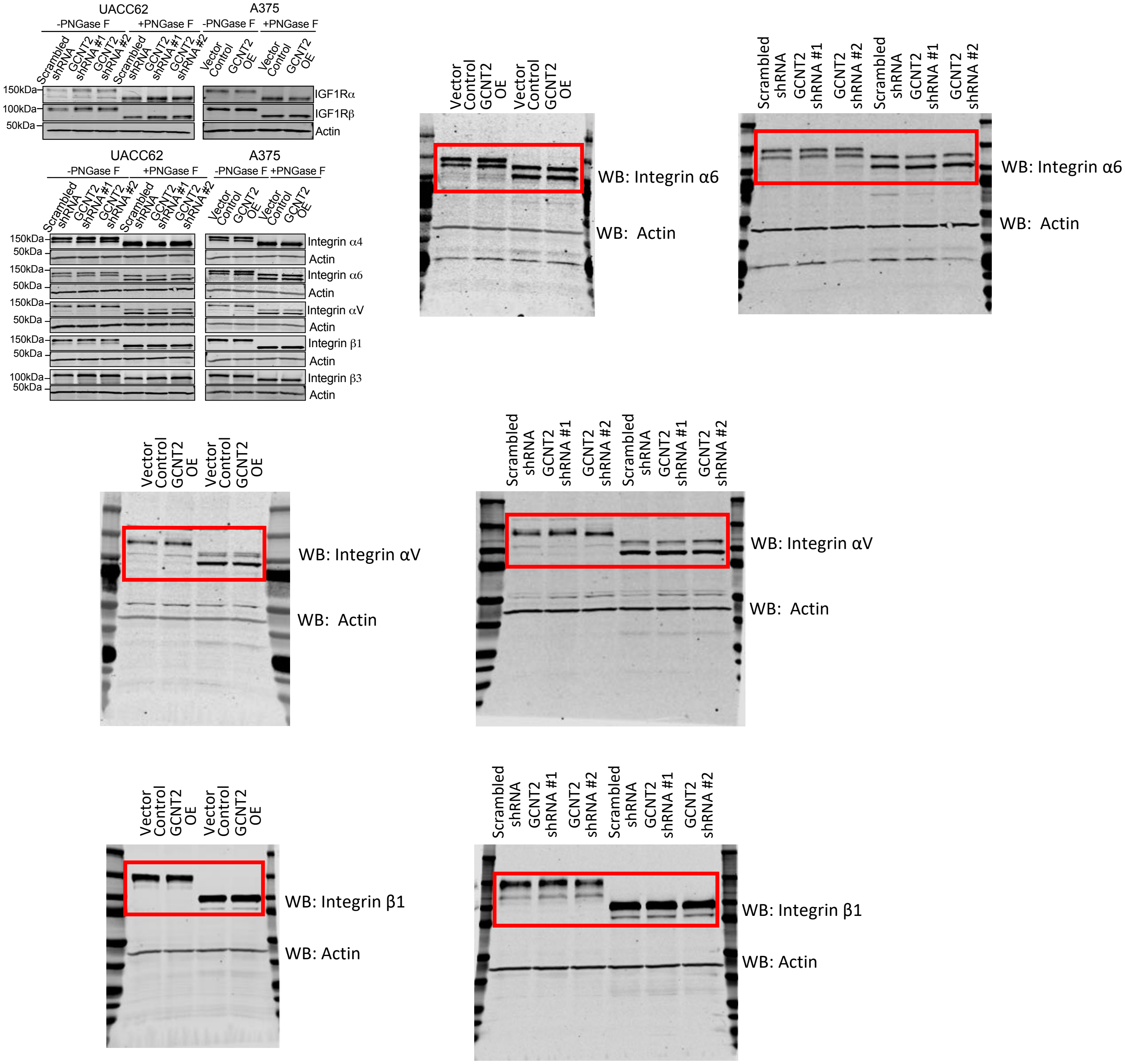

Supplementary Figure 9 (continued). Uncropped blot images From Supplemental Figure 5c

From Supplementary Figure 7h

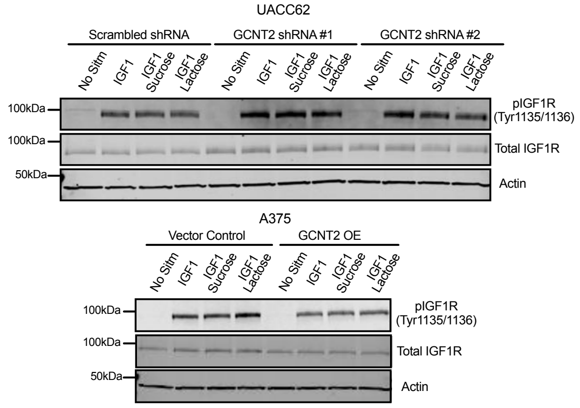

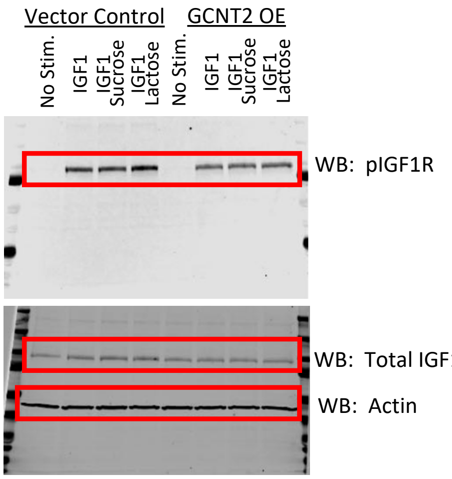

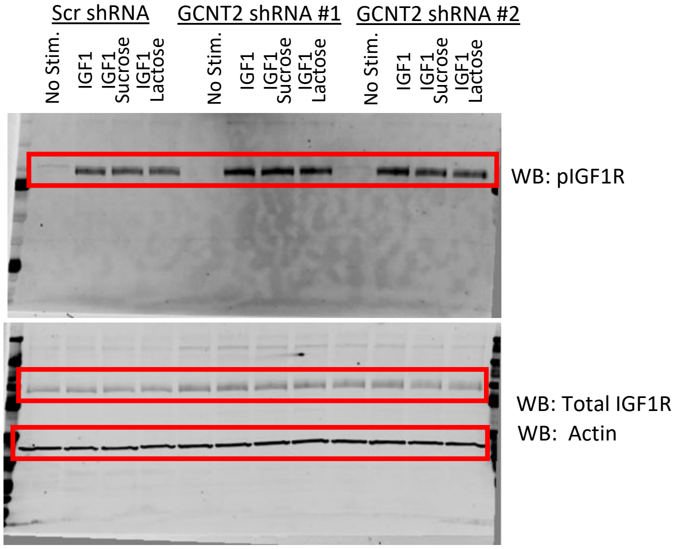

From Supplementary Figure 7j

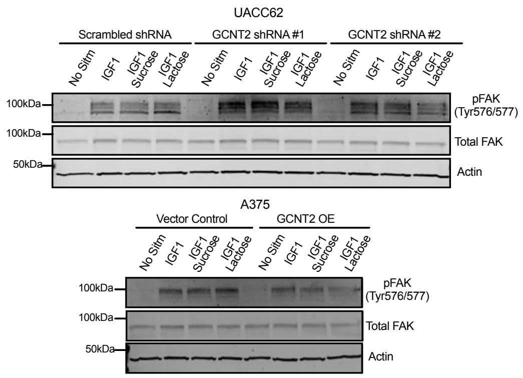

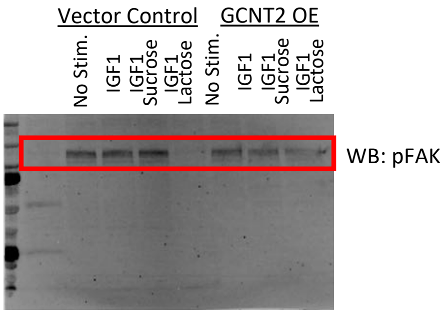

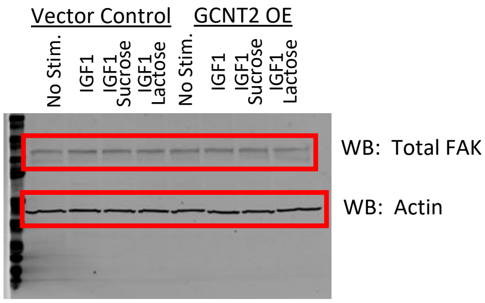

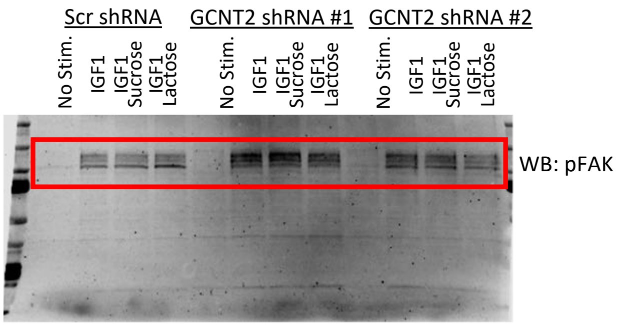

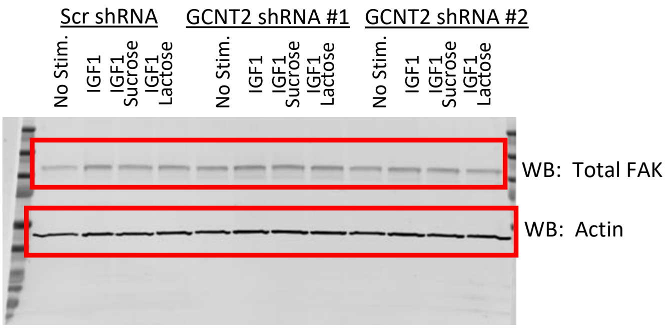

Supplementary Figure 9 (continued). Uncropped blot images From Supplemental Figure 7h and 7j

From Supplementary Figure 8a and 8c

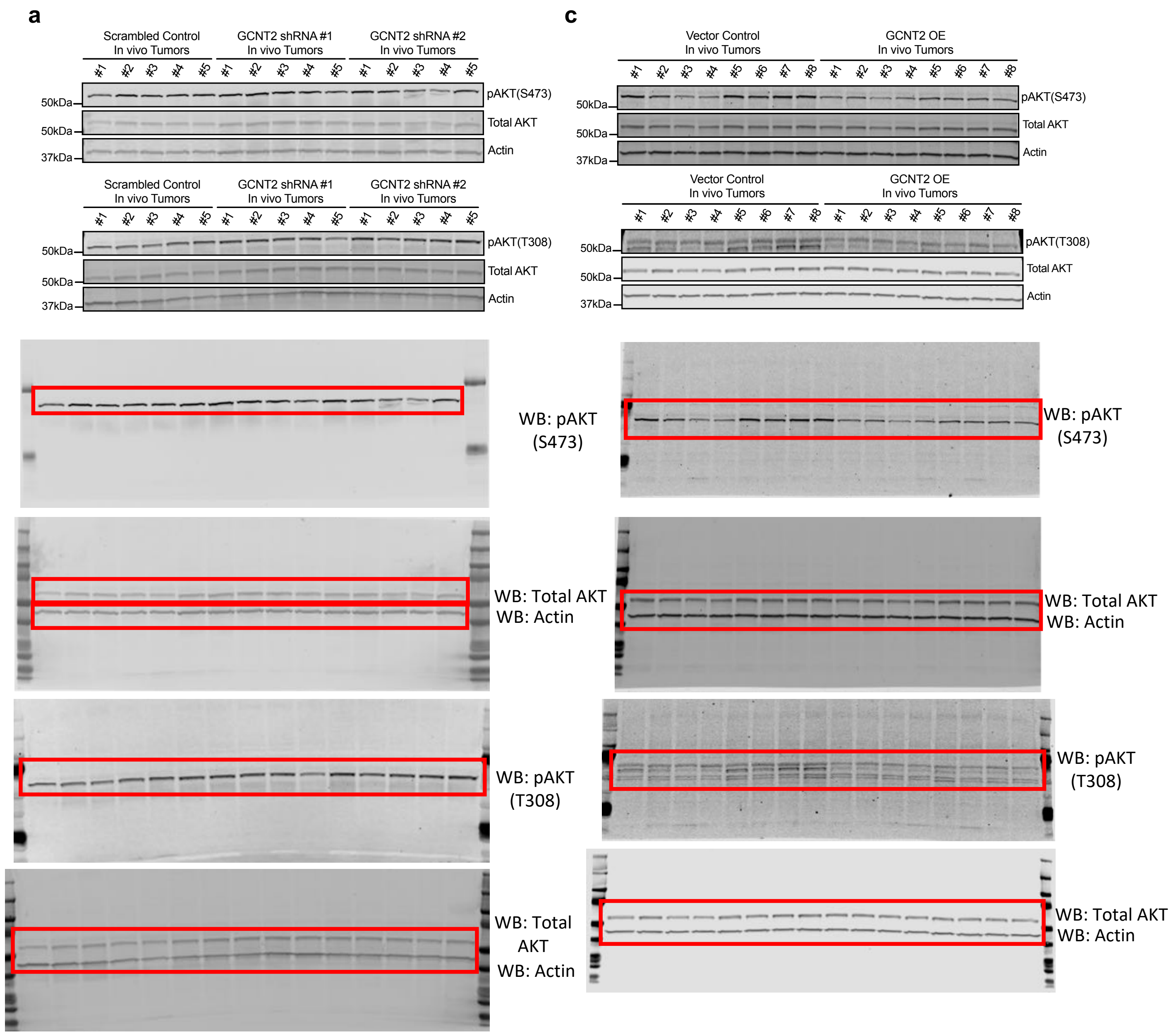

Supplementary Figure 9 (continued). Uncropped blot images From Supplemental Figure 8a and 8c

From Supplementary Figure 8e, 8h, 8k and 8n

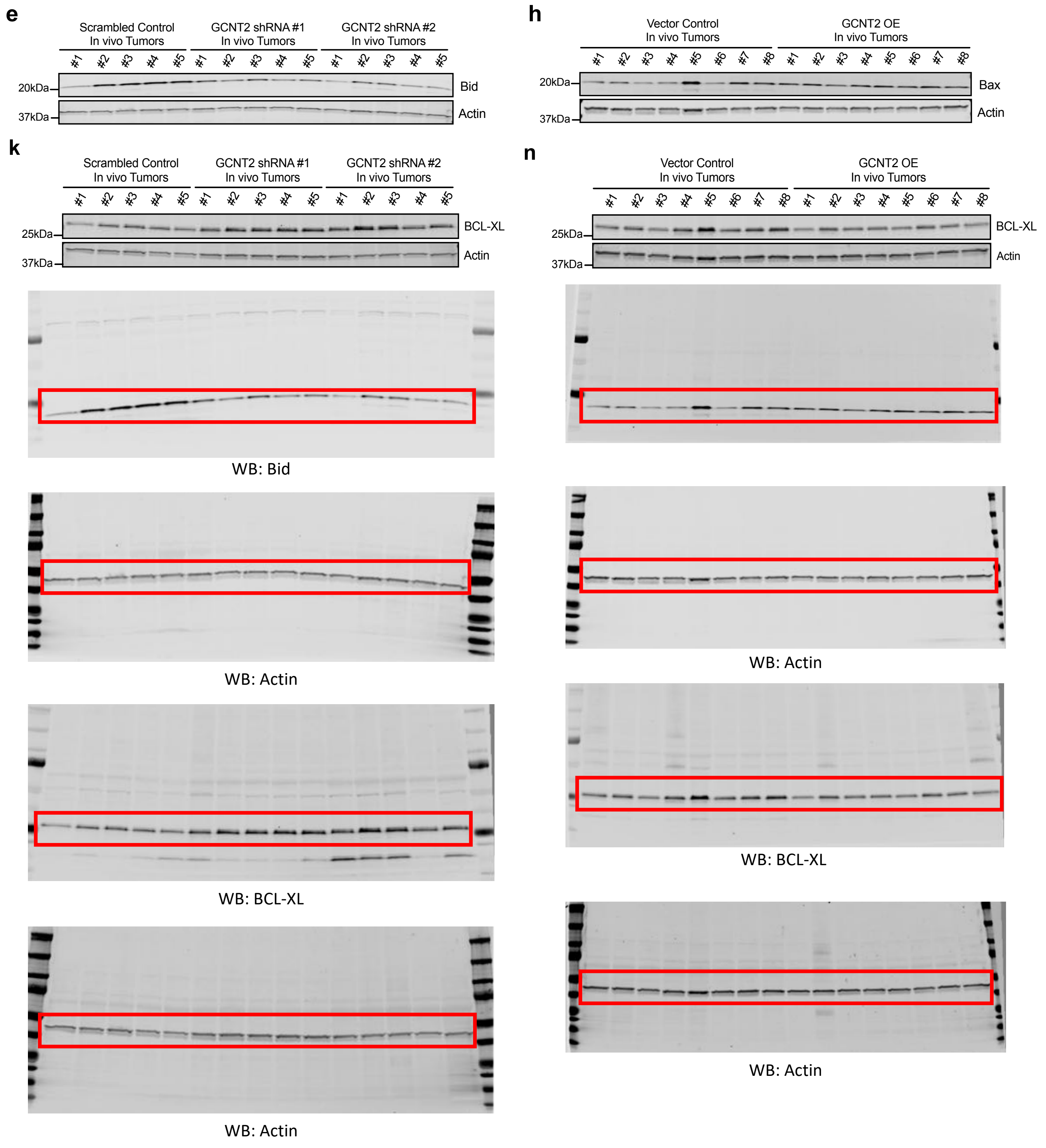

Supplementary Figure 9 (continued). Uncropped blot images From Supplemental Figure 8e, 8h, 8k and 8n

Supplementary Table 1 Oligonucleotide sequences.

Gene	Forward Sequence	Reverse Sequence	Comments
Human GCNT2	ATGCATCCTGGACTGGAAAC	AGCAAACAGGCTTGGTGAAT	Real time qPCR
Human ITGA4	GTCCTTGTTTAATGCTGGAGATGAT	GTCCTTGTTTAATGCTGGAGATGAT	Real time qPCR
Human ITGA6	GCTCGAGGTTATGGAACAGC	GCAGCAGCAGTCACATCAAT	Real time qPCR
Human ITGAV	GGAGCAATTCGACGAGCACT	TTCATCCCGCAGATACGCTA	Real time qPCR
Human ITGB1	GTAACCAACCGTAGCAAAGGAACAGC	ATGTCTGTGGCTCCCCTGATCTTA	Real time qPCR
Human ITGB3	TTCAATGCCACCTGCCTCAA	TTGGCCTCAATGCTGAAGCTC	Real time qPCR
Human IGF1R	ACAACTACGCCCTGGTCATC	CATTCCTTTGGGGGCTTATT	Real time qPCR
Human BCL-2	GTCTGGGAATCGATCTGGAA	AATGCATAAGGCAACGATCC	Real time qPCR
Human BCL-XL	TCCTCTCCCGACCTGTGATA	AGCCAAGATAAGATTCTGAAGGG	Real time qPCR
Human MCL-1	CCAAGAAAGCTGCATCGAACCAT	CAGCACATTCCTGATGCCACCT	Real time qPCR
Human BAX	ACCAAGAAGCTGAGCGAGTG	CAGTTGAAGTTGCCGTCAGA	Real time qPCR
Human BID	AAGAAGGTGGCCAGTCACAC	GTCCATCCCATTTCTGGCTA	Real time qPCR
Human BAD	CCAACCTCTGGGCAGCACAGC	TTTGCCGCATCTGCGTTGCTGT	Real time qPCR
Human B2M	TTTCATCCATCCGACATTGA	CGGCAGGCATACTCATCTTT	Real time qPCR
Human ACTB	AGAGCTACGAGCTGCCTGAC	AGCACTGTGTTGGCGTACAG	Real time qPCR
Human GCNT2	CGACAGATCTGCCACCATGAACTTTTGGAGGTACTGCTTT	TGTCAAGCTTTCAAAAATACCAGCTGGGTTGTA	Cloning primers
Human GCNT2 shRNA #1	GCTAACAAGTTTGAGCTTAAT	-	shRNA target sequence
Human GCNT2 shRNA #2	GCTCACCTCTATATTAGTTTA	-	shRNA target sequence

Supplementary Table 2 Antibodies and reagents used for flow cytometry, western blotting, and immunohistochemistry.

Antibodies / Reagents	Source	Identifier	Concentrations
Biotinylated Phaseolus vulgaris Leucoagglutinin (PHA-L) lectin	Vector Laboratories	Cat# B-1115	0.5μg/ml (FACS)
Biotinylated Sambucus Nigra Lectin (SNA)	Vector Laboratories	Cat# B-1035	0.5μg/ml (FACS)
Biotinylated Maackia Amurensis Lectin II (MAL II)	Vector Laboratories	Cat# B-1265	0.5μg/ml (FACS)
Biotinylated Solanum Tuberosum (Potato) Lectin (STA)	Vector Laboratories	Cat# B-1165	0.5μg/ml (FACS)
Biotinylated Lycopersicon Esculentum (Tomato) Lectin (LEA)	Vector Laboratories	Cat# B-1175	0.5μg/ml (FACS)<br>2μg/ml (Western)
Mouse monoclonal anti-Ganglioside GD3 (clone R24)	Abcam	Cat# ab11779	5μg/ml (FACS)
PE anti-human CD221 (IGF-1R) Antibody (clone 1H7/CD221)	Biolegend	Cat# 351805	1μg/ml (FACS)
APC anti-human CD29 (β1 integrin) (clone TS2/16)	Biolegend	Cat# 303008	0.5μg/ml (FACS)
APC anti-human CD61 (β3 integrin) (clone VI-PL2)	Biolegend	Cat# 336411	1.5μg/ml (FACS)
APC anti-human CD49d (α4 integrin) (clone 9F10)	Biolegend	Cat# 304307	0.5μg/ml (FACS)
APC anti-human CD49f (α6 integrin) (clone GoH3)	Biolegend	Cat# 313615	0.1μg/ml (FACS)
AF647 anti-human CD51 (αV integrin) (clone P1F6)	Biolegend	Cat# 920005	1.5μg/ml (FACS)
APC mouse IgG1, k isotype ctrl (clone MOPC-21)	Biolegend	Cat# 400120	0.5μg/ml (FACS)
APC ratIgG2a, k isotype ctrl (clone RTK2758)	Biolegend	Cat# 400511	1.5μg/ml (FACS)
AF647 mIgG1, k isotype ctrl (clone MOPC-21)	Biolegend	Cat# 400130	1.5μg/ml (FACS)
Rabbit monoclonal anti-phospho-Tyrosine (clone P-Tyr-1000)	Cell Signaling Technology	Cat# 8954	1:1000 (Western)
Rabbit monoclonal anti-phospho-IGF-1-Receptor β (Tyr1135/1136) (clone 19H7)	Cell Signaling Technology	Cat# 3024	1:1000 (Western)
Rabbit monoclonal anti-phospho-AKT (Ser473) (clone D9E)	Cell Signaling Technology	Cat# 4060	1:2000 (Western)
Rabbit monoclonal anti-phospho-AKT (Thr308) (clone D25E6)	Cell Signaling Technology	Cat# 13038	1:1000 (Western)
Rabbit monoclonal anti-IGF-1 Receptor β (clone D23H3)	Cell Signaling Technology	Cat# 9750	1:1000 (Western)
Mouse monoclonal anti-AKT (pan) (clone 40D4)	Cell Signaling Technology	Cat# 2920	1:2000 (Western)
Rabbit polyclonal anti-phospho-FAK (Tyr576/577)	Cell Signaling Technology	Cat# 3281	1:1000 (Western)
Rabbit monoclonal anti-phospho-p44/42 MAPK (Erk1/2) (Thr202/Tyr204) (clone D13.14.4E)	Cell Signaling Technology	Cat# 4370	1:2000 (Western)
Rabbit monoclonal anti-FAK (clone D2R2E)	Cell Signaling Technology	Cat# 13009	1:1000 (Western)

Supplementary Table 2 (continued). Antibodies and reagents used for flow cytometry, western blotting, and immunohistochemistry.

Mouse monoclonal anti-ERK1/ERK2 (clone 216703)	R&D Systems	Cat# MAB1576	0.5μg/ml (Western)
Rabbit monoclonal anti-Integrin α4 (clone D2E1)	Cell Signaling Technology	Cat# 8440	1:1000 (Western) 1:100 (IP)
Rabbit polyclonal anti-Integrin α6	Cell Signaling Technology	Cat# 3750S	1:1000 (Western)
Rabbit polyclonal anti-Integrin αV	Cell Signaling Technology	Cat# 4711S	1:1000 (Western)
Rabbit monoclonal anti-Integrin β1 (clone D2E5)	Cell Signaling Technology	Cat# 9699S	1:1000 (Western)
Rabbit monoclonal anti-Integrin β3 (clone D7X3P)	Cell Signaling Technology	Cat# 13166	1:1000 (Western)
Mouse monoclonal anti-IGF-1Rα (clone G-5)	Santa Cruz Biotechnology	Cat# sc-271606	1:200 (Western)
Mouse monoclonal anti-Actin (clone C4)	BD Biosciences	Cat# 612656	0.5μg/ml (Western)
Mouse monoclonal anti-human CD29 (β1 integrin) (clone TS2/16)	Biolegend	Cat# 303001	10μg/ml (IP)
Mouse monoclonal anti-human β3 integrin (clone D-11)	Santa Cruz	Cat# sc-365679	5μg/ml (IP)
Mouse anti-human IGF1R (clone alphaIR3)	Abcam	Cat# ab16890	2μg/ml (IP)
Rabbit IgG isotype	GeneTex	Cat# GTX35035	20μg/ml (IP)
Mouse IgG1 isotype	Biolegend	Cat# 400102	10μg/ml (IP)
Rabbit polyclonal anti-GCNT2	Sigma-Aldrich	Cat# HPA026776	1:500 (IHC)
OSK-14 (anti-I) human IgM	Dr. Yoshihiko Tani Japanese Red Cross Kinki Block Blood Center	Cat# N/A	1:200 – 1:400 (batch specific - FACS)
OSK-28 (anti-i) human IgM	Dr. Yoshihiko Tani Japanese Red Cross Kinki Block Blood Center	Cat# N/A	1:10 – 1:50 (batch specific - FACS)
IRDye® 800CW Goat anti-Rabbit IgG (H + L)	LI-COR Biosciences	Cat# 926-32211	1:15,000 (Westerns)
IRDye® 800CW Goat anti-Mouse IgG (H + L)	LI-COR Biosciences	Cat# 926-32210	1:15,000 (Westerns)
IRDye® 680RD Goat anti-Mouse IgG (H + L)	LI-COR Biosciences	Cat# 926-68070	1:15,000 (Westerns)
IRDye® 680LT Donkey anti-Rabbit IgG (H + L)	LI-COR Biosciences	Cat# 926-68023	1:15,000 (Westerns)
Anti-Microphthalmia Transcription Factor Monoclonal Antibody	Leica Biosystems	Cat# NCL-L-MITF	1:50 (IHC)
7-AAD Viability Staining Solution	Biolegend	Cat# 420403	1:200 (FACS)
Zombie NIR™ Fixable Viability Kit	Biolegend	Cat # 423105	1:400 (FACS)
APC anti-human IgM Antibody (clone MHM-88)	Biolegend	Cat# 314510	1:50 (FACS)
APC Streptavidin	Biolegend	Cat# 405207	1:400 (FACS)
PE mIgG1, k isotype ctrl (clone MOPC-21)	Biolegend	Cat# 400111	1μg/ml (FACS)
